# Regulation of Alternative Polyadenylation Events by PABPC1 Affects Erythroid Progenitor Cell Expansion

**DOI:** 10.1093/gpbjnl/qzaf116

**Published:** 2025-11-25

**Authors:** Yanan Li, Yanbo Yang, Bin Hu, Zi Wang, Wei Wang, Xiaofeng He, Xusheng Wu, Sheng Lin, Narla Mohandas, Hong Liu, Jing Gong, Long Liang, Jing Liu

**Affiliations:** Department of Hematology, the Second Xiangya Hospital; Molecular Biology Research Center, Hunan Province Key Laboratory of Basic and Applied Hematology, School of Life Sciences, Central South University, Changsha 410013, China; Department of Radiology, The Third Xiangya Hospital of Central South University, Changsha 410013, China; Hubei Key Laboratory of Agricultural Bioinformatics, College of Informatics, Huazhong Agricultural University, Wuhan 430070, China; Department of Hematology, the Second Xiangya Hospital; Molecular Biology Research Center, Hunan Province Key Laboratory of Basic and Applied Hematology, School of Life Sciences, Central South University, Changsha 410013, China; Department of Hematology, the Second Xiangya Hospital; Molecular Biology Research Center, Hunan Province Key Laboratory of Basic and Applied Hematology, School of Life Sciences, Central South University, Changsha 410013, China; Department of Radiology, The Third Xiangya Hospital of Central South University, Changsha 410013, China; Shenzhen Health Development Research and Data Management Center, Shenzhen 518000, China; Shenzhen Health Development Research and Data Management Center, Shenzhen 518000, China; Shenzhen Health Development Research and Data Management Center, Shenzhen 518000, China; Research Laboratory of Red Cell Physiology, New York Blood Center, New York, NY 10065, USA; Department of Dermatology, Xiangya Hospital, Central South University, Changsha 410008, China; Hunan Key Laboratory of Skin Cancer and Psoriasis, Hunan Engineering Research Center of Skin Health and Disease, Xiangya Clinical Research Center for Cancer Immunotherapy, Furong Laboratory, Central South University, Changsha 410008, China; Hubei Key Laboratory of Agricultural Bioinformatics, College of Informatics, Huazhong Agricultural University, Wuhan 430070, China; College of Biomedicine and Health, Huazhong Agricultural University, Wuhan 430072, China; Department of Hematology, the Second Xiangya Hospital; Molecular Biology Research Center, Hunan Province Key Laboratory of Basic and Applied Hematology, School of Life Sciences, Central South University, Changsha 410013, China; Department of Hematology, the Second Xiangya Hospital; Molecular Biology Research Center, Hunan Province Key Laboratory of Basic and Applied Hematology, School of Life Sciences, Central South University, Changsha 410013, China

**Keywords:** PABPC1, TSC22D1, Alternative polyadenylation, Erythropoiesis, Erythroid progenitor

## Abstract

Erythropoiesis is precisely regulated by multilayered networks. It is crucial for maintaining steady-state hemoglobin levels and ensuring effective oxygen transport. Alternative polyadenylation (APA) is a post-transcriptional regulatory mechanism generating multiple mRNA isoforms from a single gene based on specific 3′ untranslated region sequences. While APA plays a vital role in various cellular processes, the underlying mechanism in erythropoiesis remains largely unexplored. In this study, we employed an integrative approach, combining bioinformatics analyses and experimental validations, to systematically investigate the role of APA in erythropoiesis. We mapped the APA landscape during erythroid differentiation and identified significant APA shifts essential for the differentiation of erythroid cells from burst-forming unit erythroid (BFU–E) to colony-forming unit erythroid (CFU–E). Notably, our findings highlighted polyadenylate-binding protein cytoplasmic 1 (PABPC1) as the primary regulator of APA during these stages. Functional analyses have revealed that knockdown of *PABPC1* disrupts erythroid progenitor cell proliferation and differentiation. These results implicate an essential role of PABPC1 in modulating cell fate through APA regulation. Furthermore, we found that decreased PABPC1 levels increased the usage of the proximal polyadenylation sites in the TSC22 domain family member 1 (*TSC22D1*) gene. This shift led to elevated expression of *TSC22D1*, uncovering a novel mechanism by which APA influences erythroid progenitor expansion and differentiation. Our findings provide novel insights into APA regulation in early erythropoiesis and suggest potential therapeutic strategies for diseases associated with erythropoietic disorders.

## Graphical abstract

**Figure:**
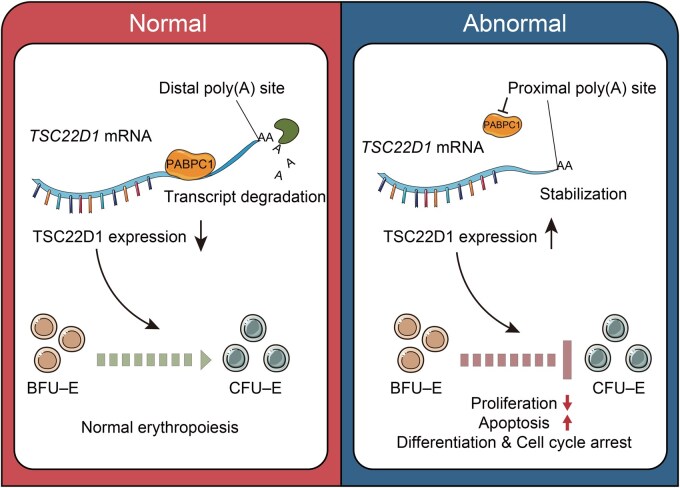

## Introduction

Hematopoietic stem cells (HSCs) generate megakaryocyte/erythrocyte progenitors through lineage commitment [1,2]. These progenitors differentiate into erythroid progenitors known as burst-forming unit erythroid (BFU–E) and colony-forming unit erythroid (CFU–E) [3,4]. Subsequently, erythroid progenitors undergo significant developmental changes and ultimately mature into red blood cells [3,5]. The expansion of erythroid progenitor cells significantly limits the efficacy of *in vitro* erythropoiesis induction [6]. Dysregulated proliferation is closely associated with various hematological disorders. For example, reduced expansion of erythroid progenitor cells has been observed in Diamond-Blackfan anemia (DBA) and is recognized as a critical factor contributing to erythropoietin-unresponsive anemia [7]. Meanwhile, abnormal proliferation has been reported to be associated with diseases such as myelodysplastic syndromes (MDS), polycythemia vera (PV), and thalassemia [8–10]. Prior studies have identified multiple molecules and pathways involved in regulating erythroid progenitor cell expansion. For instance, glucocorticoids promote BFU–E expansion, whereas inhibition of the TGF-β pathway and increased expression of *ZFP36L2* enhance BFU–E proliferation [11,12]. Despite these advances, the specific mechanisms underlying erythroid progenitor cell expansion and their regulation remain incompletely understood [11].

Alternative polyadenylation (APA) occurs through the interactions of specific RNA-protein complexes and several key *cis*-elements, such as polyadenylation [poly(A)] signals around the different poly(A) sites (PASs). APA generates transcript isoforms with diverse 3′ untranslated regions (3′ UTRs) [13–15]. Previous studies have indicated that approximately 50%–80% of mammalian pre-mRNAs harbor multiple PASs [16,17]. The widespread occurrence of APA not only enhances transcript complexity but also results in the gain or loss of binding sites for RNA-binding proteins (RBPs) and microRNAs (miRNAs). Consequently, APA influences mRNA stability, localization, and translation [13,14,16,18].

Over the past few years, researchers have extensively studied the regulation of APA in various biological processes. These processes include neuronal differentiation, cell proliferation, and immune cell activation [13,14,16–24]. For instance, in breast cancer, APA-mediated shortening of the 3′ UTRs of *NRAS* and *c-JUN* has been observed [25,26]. Tumors harboring these shortened mRNA isoforms exhibit lower proliferation rates but increased invasiveness [25,26]. In the context of cell proliferation, increased cellular activity has been associated with widespread 3′ UTR shortening [25,26]. This shortening is an integral part of the gene expression program involved in cell proliferation, differentiation, and development [27]. Core APA factors significantly influence this process. For example, PABPN1, a critical regulator of APA, has been shown to regulate the genome-wide APA dynamics in HSCs [28].

Research on APA in erythropoiesis is less extensive, with only a few APA-related RBP studies reported [29,30]. One example is the APA core regulator PABPC4, which has been identified to bind to and stabilize the transcript of *GPA*, along with other erythroid targets such as *HBA1/HBA2*,* HBB*,* BTG2*, and *SLC4A1* [29]. These interactions are critical for maintaining terminal erythroid maturation [29]. However, the global APA patterns during erythroid differentiation and their functional roles in the early stages of erythropoiesis remain largely unexplored.

In this study, we employed a combination of bioinformatics analyses and experiments to examine global APA patterns across different stages of erythropoiesis. This approach enabled us to precisely capture and map the dynamic APA landscape during erythroid differentiation, pinpointing key APA shifts pivotal for the transition from BFU–E to CFU–E stages. Our findings revealed a marked downregulation of the APA factor polyadenylate-binding protein cytoplasmic 1 (PABPC1) during these stages, which likely precipitates notable APA alterations. Furthermore, we identified TSC22 domain family member 1 (*TSC22D1*) as a crucial target influenced by PABPC1-mediated APA events. Changes in PABPC1 levels promoted the usage of proximal PASs (pPASs), resulting in increased expression of *TSC22D1*. In-depth analysis demonstrated that *TSC22D1* plays a crucial role in the expansion and differentiation of erythroid progenitor cells. Overall, these discoveries clarify the molecular mechanisms of APA at early stages of erythropoiesis and underscore potential therapeutic targets for disorders involving abnormal erythroid cell development.

## Results

### Global APA patterns in erythropoiesis

**Figure 1:**
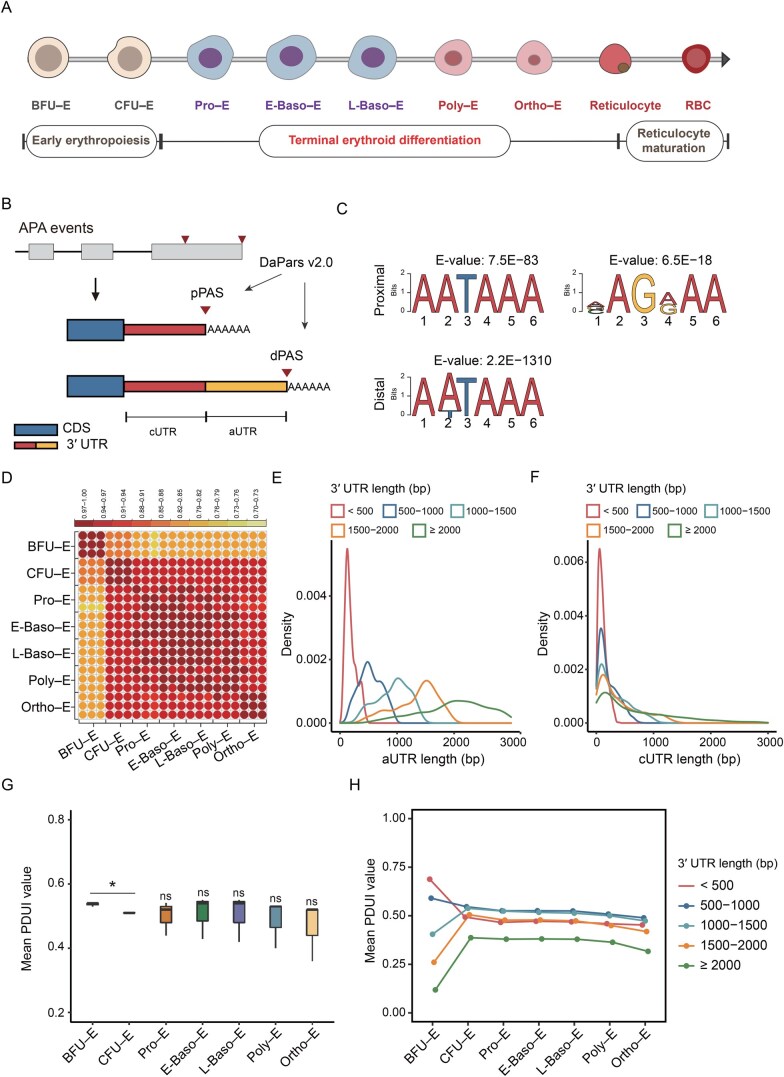
Global APA patterns in erythropoiesis **A**. Schematic diagram of different stages of human erythroid differentiation. The development process of erythropoiesis includes three stages: early erythropoiesis, terminal erythroid differentiation, and reticulocyte maturation. **B**. Schematic of APA and alternative polyadenylation isoforms using pPAS or dPAS in the 3′ UTR. The region between the PASs is named aUTR. The common region of isoforms is named cUTR. **C**. Enriched motifs around pPASs and dPASs of APA events. **D**. Pearson’s correlation heatmap displaying the distal PDUI across erythropoiesis stages. The color of each cell represents the correlation coefficient, with dark red signifying a strong positive correlation and yellow denoting a weaker correlation. **E**. Density distribution of aUTR lengths across various 3′ UTR length categories. The color indicates the stratification of 3′ UTR lengths into different categories. **F**. Density distribution of cUTR lengths across various 3′ UTR length categories. The color indicates the stratification of 3′ UTR lengths into different categories. **G**. The average relative 3′ UTR length at each stage of human erythropoiesis (BFU–E to Ortho–E). Statistical significance was determined by the Wilcoxon signed-rank test (*, *P* < 0.05; ns, not significant). **H**. Mean PDUI values across different erythroid maturation stages for 3′ UTR length categories. The x-axis indicates the erythroid maturation stages from BFU–E to Ortho–E, while the y-axis shows the mean PDUI values. Each line represents a distinct 3′ UTR length category. APA, alternative polyadenylation; BFU–E, burst-forming unit erythroid; CFU–E, colony-forming unit erythroid; Pro–E, proerythroblast; E-Baso–E, early basophilic erythroblast; L-Baso–E, late basophilic erythroblast; Poly–E, polychromatic erythroblast; Ortho–E, orthochromatic erythroblast; RBC, red blood cell; PAS, polyadenylation site; pPAS, proximal polyadenylation site; dPAS, distal polyadenylation site; UTR, untranslated region; aUTR, alternative 3′ UTR; cUTR, common 3′ UTR; PDUI, polyadenylation site usage index.

Efficient regulation of erythropoiesis is crucial for maintaining systemic homeostasis [7–10]. To explore APA’s influence on erythroid differentiation, we retrieved RNA sequencing (RNA-seq) data from our previous research, which includes both early (GSE61566) and late (GSE53983) stages of erythropoiesis. These datasets span seven phases, ranging from BFU–E to orthochromatic erythroblasts (**Figure 1**A). We further employed DaPars (v2.0) to systematically estimate the relative usage of APA on these datasets [31]. DaPars (v2.0) utilizes a two-normal mixture model to quantify APA usage across multiple samples (Figure 1B). In total, we identified 8377 APA events during erythropoiesis. Poly(A) signal motifs are key *cis*-elements involved in poly(A) and play crucial roles in mRNA processing and stabilization [32,33]. To investigate the preferred poly(A) signal motifs of these APA events, we extracted sequences of ± 50 nt around each pPASs and distal PASs (dPASs). We then employed DREME to enrich poly(A) signal features. Our study revealed that the dPASs preferentially utilized canonical poly(A) signal motifs AATAAA and ATTAAA, while pPASs showed a preference for AATAAA and AAGAAA; notably, AAGAAA is recognized as an active poly(A) signal in mammalian cells [34] (Figure 1C). To validate the biological reproducibility of distal poly(A) site usage index (PDUI) values, we conducted Pearson’s correlation analyses on the PDUI matrix across erythropoiesis. We observed clear stage-specific clustering, indicating significant variations in PAS utilization across different erythropoiesis stages (Figure 1D).

We further investigated the structural characteristics of APA events during erythropoiesis. The 3′ UTR lengths of genes undergoing APA events were significantly longer compared to other genes (Wilcoxon test, *P* < 2.2E−16) (Figure S1A). To explore the finer structural differences among various 3′ UTR lengths, we categorized these lengths into distinct bins (< 500 bp, 500–1000 bp, 1000–1500 bp, 1500–2000 bp, ≥ 2000 bp). APA events were predominantly located within shorter 3′ UTR length bins, particularly those shorter than 500 bp (Figure 1E and F). The alternative 3′ UTR (aUTR) is defined as the portion of the 3′ UTR that exhibits length variations, whereas the common 3′ UTR (cUTR) remains consistent across different isoforms [35]. We observed that longer 3′ UTRs correlated with longer aUTRs (Figure 1E, Figure S1B), whereas the cUTR remained relatively stable across different bin categories (Figure 1F, Figure S1C). The 3′ UTRs contain a variety of *cis*-elements, such as miRNA target sites and RBP binding sites. Longer 3′ UTRs and aUTRs imply an increased variability of these regulatory elements, potentially influencing gene expression and cellular functions.

Next, we investigated APA dynamics during erythropoiesis. We observed a decrease in PDUI during the early phases of erythropoiesis (Figure 1G), suggesting enhanced utilization of pPASs from BFU–E to CFU–E stages. Additionally, APA events with shorter 3′ UTRs (< 1000 bp) exhibited increased utilization of pPASs, whereas those with longer 3′ UTRs preferentially transitioned toward dPASs selection (Figure 1H). These findings highlight the complex regulatory role of APA and underscore its potential biological significance in early erythroid progenitor cells.

### Functional diversity of APA dynamics in erythropoiesis phases associated with cell cycle and RNA regulation

**Figure 2:**
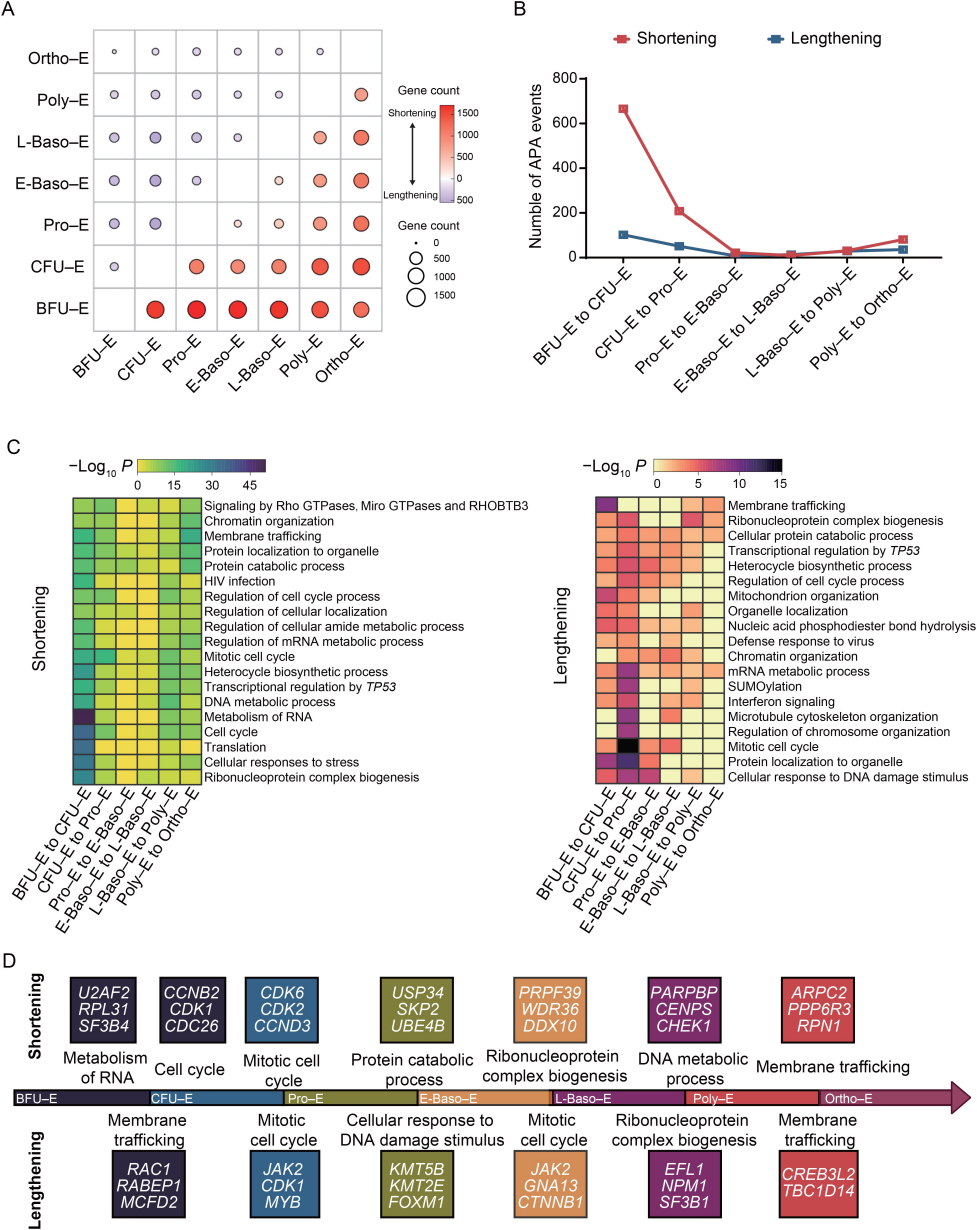
Functional diversity of APA dynamics in erythropoiesis phases associated with cell cycle and RNA regulation **A**. The heatmap shows the number of genes with 3′ UTR length changes (including lengthening and shortening) at different erythroid differentiation stages, from BFU–E to Ortho–E. **B**. The line plot displays the variation in the number of APA events from BFU–E to Ortho–E stages, where the red line indicates the number of genes with shortened 3′ UTRs, and the blue line shows the number of genes with lengthened 3′ UTRs. **C**. The heatmap illustrates gene enrichment results for APA patterns (shortening and lengthening events) during erythropoiesis. The left panel lists biological processes enriched among genes exhibiting 3′ UTR shortening. The right panel lists biological processes enriched among genes exhibiting 3′ UTR lengthening. **D**. Example diagram of enriched pathway-related genes in various stages of erythropoiesis. The upper panel lists key genes associated with 3′ UTR shortening and their related biological processes. The lower panel lists key genes associated with 3′ UTR lengthening and their related biological processes. *U2AF2*, U2 small nuclear RNA auxiliary factor 2; *RPL31*, ribosomal protein L31; *SF3B4*, splicing factor 3b subunit 4; *CCNB2*, cyclin B2; *CDK1*, cyclin-dependent kinase 1; *CDC26*, cell division cycle 26; *CDK6*, cyclin-dependent kinase 6; *CDK2*, cyclin-dependent kinase 2; *CCND3*, cyclin D3; *USP34*, ubiquitin specific peptidase 34; *SKP2*, S-phase kinase associated protein 2; *UBE4B*, ubiquitination factor E4 B; *PRPF39*, pre-mRNA processing factor 39; *WDR36*, WD repeat domain 36; *DDX10*, DEAD-box helicase 10; *PARPBP*, PARP binding protein; *CENPS*, centromere protein S; *CHEK1*, checkpoint kinase 1; *ARPC2*, actin related protein 2/3 complex subunit 2; *PPP6R3*, protein phosphatase 6 regulatory subunit 3; *RPN1*, ribophorin I; *RAC1*, Rac family small GTPase 1; *RABEP1*, RAB GTPase effector protein 1; *MCFD2*, multiple coagulation factor deficiency 2; *MYB*, V-myb avian myeloblastosis viral oncogene homolog; *KMT5B*, lysine methyltransferase 5B; *KMT2E*, lysine methyltransferase 2E; *FOXM1*, forkhead box M1; *JAK2*, Janus kinase 2; *GNA13*, G protein subunit alpha 13; *CTNNB1*, catenin beta 1; *EFL1*, elongation factor like GTPase 1; *NPM1*, nucleophosmin 1; *SF3B1*, splicing factor 3b subunit 1; *CREB3L2*, cAMP responsive element binding protein 3-like 2; *TBC1D14*, TBC1 domain family member 14.

To investigate genes undergoing significant changes in 3′ UTR length during erythropoiesis, we compared PDUI values between different stages. We identified 5435 genes exhibiting either 3′ UTR lengthening or shortening. We observed a pronounced trend in which APA events involving 3′ UTR shortening significantly outnumbered those showing 3′ UTR lengthening. These APA alterations predominantly occurred during the transition from the BFU–E to Pro–E stages and stabilized during late erythropoiesis (**Figure 2**A and B). To elucidate the functional roles of these genes, we categorized them according to their APA types (3′ UTR lengthening or shortening) and conducted gene enrichment analysis. We found that APA events at different stages were mainly enriched in pathways associated with cell cycle and RNA regulation. Specifically, during the transition from BFU–E to CFU–E stages, APA shortening events were most significantly enriched in pathways such as “cell cycle” and “metabolism of RNA”. Conversely, during the transition from CFU–E to Pro–E stages, APA lengthening events were predominantly enriched in pathways including “mitotic cell cycle” and “protein localization to organelle”. These results indicate a dynamic interplay between APA event regulation and erythropoiesis (Figure 2C).

Next, we focused on representative APA genes previously demonstrated to be essential for erythropoiesis. During the BFU–E to CFU–E stages, the representative genes were enriched in pathways including: RNA metabolism (*U2AF2*, *RPL31*, and *SF3B4*), cell cycle (*CCNB2*, *CDK2*, and *CDC26*), and membrane transport (*RAC1*, *RABEP1*, and *MCFD2*). In the terminal stages of erythropoiesis, these genes were enriched in pathways such as: mitotic cell cycle (*CDK6*, *CDK2*, *CCND3*, *JAK2*, *CDK1*, and *MYB*), biogenesis of ribonucleoprotein complexes (*PRPF39*, *WDR36*, *DDX10*, *EFL1*, *NPM1*, and *SF3B1*), membrane transport (*ARPC2*, *PPP6R3*, *RPN1*, *CREB3L2*, and *TBC1D14*), and protein metabolic processes (*USP34*, *SKP2*, and *UBE4B*) (Figure 2D). These findings suggest that APA dynamics play diverse functional roles across different erythropoiesis phases. Particularly, early erythropoiesis stages show a marked association with cell cycle and RNA regulation.

### PABPC1 as a potential regulator of APA-mediated 3′ UTR shortening during the transition from BFU–E to CFU–E

**Figure 3:**
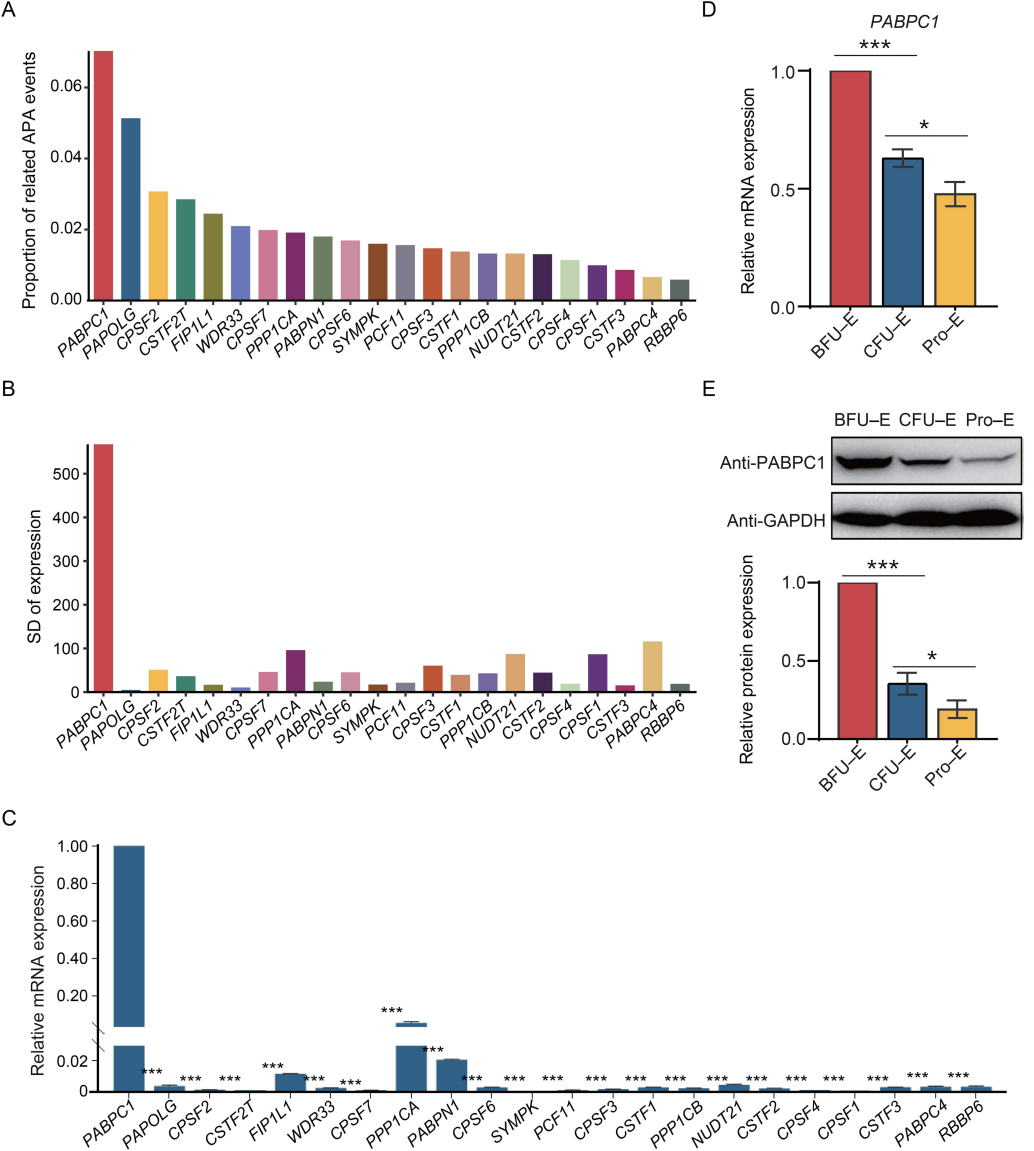
PABPC1 as a potential regulator of APA-mediated 3′ UTR shortening during the transition from BFU–E to CFU–E **A**. The bar chart shows the ability of APA regulatory factors to regulate APA events during erythroid development. The vertical axis represents the proportion of APA-3′ end processing factor associations relative to all APA events with 3′ UTR lengthening or shortening during differentiation, which reflects the ability of core factors to regulate APA events. The horizontal axis represents the core APA regulatory factors. **B**. The SD of the expression levels of core APA factors across all samples of erythrocyte differentiation. **C**. qRT-PCR shows the mRNA expression of core APA factors relative to *PABPC1* in early erythropoiesis (the BFU–E stage). Statistical significance was assessed by comparing each gene’s expression to PABPC1. ***, *P* < 0.001 (Student’s *t*-test). Human mobilized peripheral blood CD34^+^ cells were induced to differentiate into early erythroid lineage cells *in vitro*, and cells at the BFU–E stage were sorted for analysis. ∗, *P* < 0.05; ∗∗∗, *P* < 0.001 (Student's *t*-test). **D**. qRT-PCR shows the mRNA expression of *PABPC1* from BFU–E to Pro–E stages. Human mobilized peripheral blood CD34^+^ cells were induced to differentiate into early erythroid lineage cells *in vitro*, and cells at the BFU–E stage were sorted for analysis. *, *P* < 0.05; ***, *P* < 0.001 (Student’s *t*-test). **E**. Representative images of Western blotting showing PABPC1 expression from BFU–E to Pro–E stages. Cells at stage from the BFU–E to Pro–E were sorted following *in vitro* differentiation of human mobilized peripheral blood CD34^+^ cells into the specific erythroid lineage. Quantitative analysis of PABPC1 expression from the BFU–E to Pro–E stages based on three independent experiments is shown. GAPDH was used as a loading control. *, *P* < 0.05; ***, *P* < 0.001 (Student’s *t*-test). *PABPC1*, polyadenylate-binding protein cytoplasmic 1; *PAPOLG*, polyadenylation polymerase gamma; *CPSF2*, cleavage and polyadenylation specific factor 2; CSTF2T, cleavage stimulation factor subunit 2T; *FIPIL1*, FIP1-like 1; *WDR33*, WD repeat domain 33; *CPSF7*, cleavage and polyadenylation specific factor 7; *PPP1CA*, protein phosphatase 1 catalytic subunit alpha; *PABPN1*, polyadenylate-binding protein nuclear 1; *CPSF6*, cleavage and polyadenylation specific factor 6; *SYMPK*, symplekin; *PCF11*, cleavage and polyadenylation factor 11; *CPSF3*, cleavage and polyadenylation specific factor 3; *CSTF1*, cleavage stimulation factor subunit 1; *PPP1CB*, protein phosphatase 1 catalytic subunit beta; *NUDT21*, nudix hydrolase 21; *CSTF2*, cleavage stimulation factor subunit 2; *CPSF4*, cleavage and polyadenylation specific factor 4; *CPSF1*, cleavage and polyadenylation specific factor 1; *CSTF3*, cleavage stimulation factor subunit 3; *PABPC4*, polyadenylate-binding protein cytoplasmic 4; *RBBP6*, retinoblastoma binding protein 6; SD, standard deviation.

3′ end processing factors regulate APA by influencing PASs selection [35–37]. These factors recognize specific PASs and determine proximal or distal site usage. Consequently, they generate diverse mRNA isoforms that affect gene expression, mRNA stability, localization, and ultimately cellular differentiation and development [35–37]. To investigate key regulators of genes exhibiting significant 3′ UTR length changes during early erythropoiesis, we first assembled a list of known core APA factors. Subsequently, we calculated the correlation between the PDUI of each APA event and the expression of each APA factor. Significant associations between known 3′ end processing factors and APA events were identified using stringent criteria (*R*^2^ ≥ 0.3 and FDR < 0.05). After aggregating these association results by 3′ end processing factors, we identified the top 10 candidate regulators of APA events: *PABPC1*,* PAPOLG*,* CPSF2*,* CSTF2T*,* FIP1L1*,* WDR33*,* CPSF7*,* PPP1CA*,* PABPN1*, and *CPSF6* (**Figure 3**A). To determine whether these factors exhibit genuine variability during differentiation — and thus a potential role in erythropoiesis — we calculated the standard deviation of their expression levels (measured in TPM) across erythroid differentiation samples. Our findings demonstrated that *PABPC1* exhibited the highest standard deviation, markedly higher than those of other factors. This observation suggests a considerable fluctuation in *PABPC1* expression throughout erythropoiesis, highlighting its potential involvement in the regulatory mechanisms that govern erythropoiesis (Figure 3B). Given the significant APA changes during early erythropoiesis, we quantified the mRNA expression levels of core APA factors during these critical phases. Notably, only *PABPC1* exhibited relatively high expression at the BFU–E stage (Figure 3C). To further validate this finding, we induced differentiation of CD34^+^ cells (isolated from human mobilized peripheral blood) into the erythroid lineage [3]. We observed that both mRNA and protein expression levels of PABPC1 peaked in the BFU–E stage and subsequently declined in the CFU–E stage (Figure 3D and E). Among the APA events significantly associated with *PABPC1*, we observed 1276 events leading to 3′ UTR shortening and 243 events resulting in 3′ UTR lengthening. This result suggests that knockdown of *PABPC1* may increase the usage of pPASs. Previous studies have demonstrated that PABPC1 promotes dPAS usage and its knockdown leads to 3′ UTR shortening [38]; however, such observations have not yet been reported in the erythroid lineage cells. Collectively, these results suggest that PABPC1 is likely a crucial APA regulator that mediates 3′ UTR shortening of transcripts during the transition from BFU–E to CFU–E stages of erythropoiesis.

### PABPC1 plays a crucial role in the transition from the BFU–E to CFU–E stage, impacting proliferation and differentiation

PABPC1 is widely expressed in most eukaryotes [39]. This factor interacts with various proteins and exhibits complex intracellular localization [40]. In mammals, PABPC1 is a multifunctional RBP, indispensable for protein translation initiation, RNA processing, RNA stability, and the promotion of dPASs utilization [39,41]. Under normal conditions, PABPC1 predominantly localizes in the cytoplasm [40,42]. However, under specific circumstances, it shuttles between the nucleus and cytoplasm. In the nucleus, it participates in polyadenylation of intron-containing precursor mRNAs by interacting with poly(A) polymerase, thereby influencing nuclear RNA processing [40,42].

**Figure 4:**
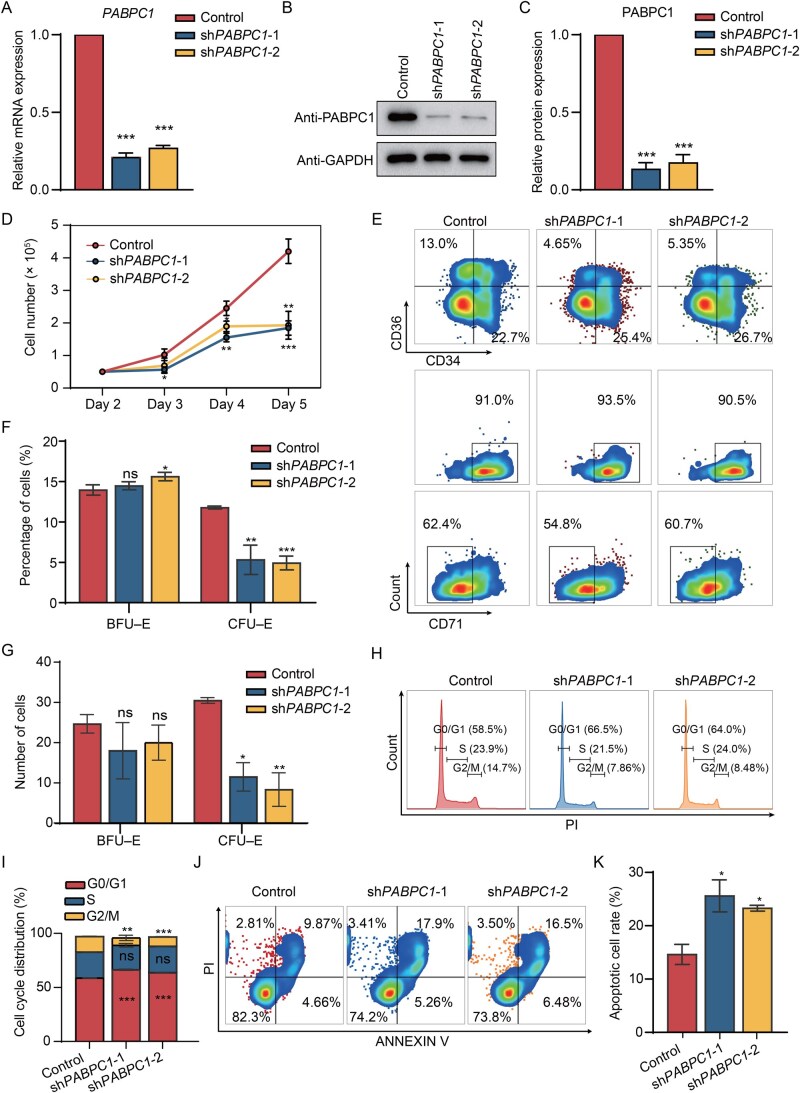
PABPC1 plays a crucial role in the transition from the BFU–E to CFU–E stage in impacting proliferation and differentiation **A**. qRT-PCR shows *PABPC1* mRNA expression in early erythroblasts infected with lentivirus containing control shRNA or *PABPC1* shRNA. ***, *P* < 0.001 (Student’s *t*-test). **B**. Representative images of Western blotting showing PABPC1 expression in erythroblasts infected with lentivirus containing control shRNA or *PABPC1* shRNA. **C**. Quantitative analysis of protein expression data from three independent experiments, as shown in (B). GAPDH was used as a loading control. ***, *P* < 0.001 (Student’s *t*-test). **D**. Early erythroid cell (BFU–E) growth curves determined by cell counting. Early erythroid cells following infection with the same amount of lentivirus as indicated in (A). Statistical analysis of the data was based on three independent experiments comparing the knockdown and control groups using Student’s *t*-test, and the bar plot shows the mean ± SD of triplicate samples. *, *P* < 0.05; **, *P* < 0.01; ***, *P* < 0.001. **E**. Representative flow cytometry data of early differentiation for early erythroid cells (BFU–E) infected with the same amount of lentivirus as indicated in (A). CD34^−^CD36^+^CD71^high^ represents the stage of CFU–E cells, while CD34^+^CD36^−^CD71^low^ represent the stage of BFU–E cells. CD34^−^CD36^+^CD71^high^ and CD34^+^CD36^−^CD71^low^ cells were gated from CD45^+^GPA^−^CD123^−^ cells. **F**. Statistical analysis of the percentages of CD34^−^CD36^+^CD71^high^ (CFU–E) and CD34^+^CD36^−^CD71^low^ (BFU–E) cells from three independent experiments is shown. *, *P* < 0.05; **, *P* < 0.01; ***, *P* < 0.001; ns, not significant (Student’s *t*-test). **G**. Statistical analysis of the number of BFU–E and CFU–E formed in soft agar clone formation experiments. *, *P* < 0.05; **, *P* < 0.01; ***, *P* < 0.001; ns, not significant (Student’s *t*-test). **H**. Representative flow cytometry data of cell cycle for early erythroid cells (BFU–E) infected with the same amount of lentivirus as indicated in (A). **I**. Statistical analysis of the PI fluorescence intensity representing each stage of the cell cycle (G0/G1, S, and G2/M) from three independent experiments is shown. *, *P* < 0.05; **, *P* < 0.01; ***, *P* < 0.001; ns, not significant (Student’s *t*-test). **J**. Representative flow cytometry data of cell apoptosis for early erythroid cell (BFU–E) infected with the same amount of lentivirus as indicated in (A). **K**. Statistical analysis of the apoptotic cell rate from three independent experiments is shown. *, *P* < 0.05; **, *P* < 0.01; ***, *P* < 0.001; ns, not significant (Student’s *t*-test). PI, propidium iodide.

To further investigate the role of PABPC1 in early human erythropoiesis, we employed a shRNA lentivirus to knock down *PABPC1* expression during initial erythroid differentiation stages *in vitro* (**Figure 4**A–C). We performed cell counting and generated growth curves to evaluate the effect of *PABPC1* knockdown on cell proliferation. Our results revealed a significant inhibition of cell proliferation following *PABPC1* knockdown (Figure 4D). Next, we employed flow cytometry to examine the impact of *PABPC1* knockdown on early erythropoiesis. The results indicated a marked decrease in the proportion of CFU–E cells (CD34^−^CD36^+^CD71^+^). However, the proportion of BFU–E cells (CD34^+^CD36^−^CD71^−^) remained largely unchanged (Figure 4E and F). Consistently, colony formation assays revealed a significant reduction in CFU–E colony numbers after *PABPC1* knockdown, whereas BFU–E colony numbers showed no significant change (Figure 4G). Additionally, we evaluated the cell cycle and apoptosis using flow cytometry (Figure 4H–K). Knockdown of *PABPC1* led to increased apoptosis and caused cell cycle arrest in the G0/G1 phase (Figure 4H–K). To further investigate the functional roles of PABPC1, we overexpressed *PABPC1* in CD34^+^ HSCs undergoing erythroid differentiation *in vitro*. Flow cytometry analysis showed that *PABPC1* overexpression significantly increased the proportion of CFU–E cells, while BFU–E cells were largely unaffected (Figure S2A and B). Additionally, colony formation assays further supported these findings, showing increased CFU–E colony numbers upon *PABPC1* overexpression. No significant change was observed in BFU–E colony numbers (Figure S2C). Given the previously reported importance of related factors (PABPN1 and PABPC4), we also measured their mRNA expression levels. Our results indicated that both *PABPN1* and *PABPC4* were expressed at relatively low levels (Figure S3A). Notably, *PABPN1* expression remained stable throughout early erythroid differentiation (Figure S3A), whereas *PABPC4* expression significantly increased from the BFU–E to CFU–E stage despite its overall low expression (Figure S3A). Based on this finding, we conducted functional studies focused on *PABPC4.* However, knockdown of *PABPC4* did not significantly affect erythroid progenitor proliferation or differentiation (Figure S3B–E). Collectively, our results highlight that PABPC1 plays a critical role in regulating the expansion and transition of erythroid progenitor cells.

### PABPC1 knockdown significantly affects APA events from BFU–E to CFU–E during early erythropoiesis

**Figure 5:**
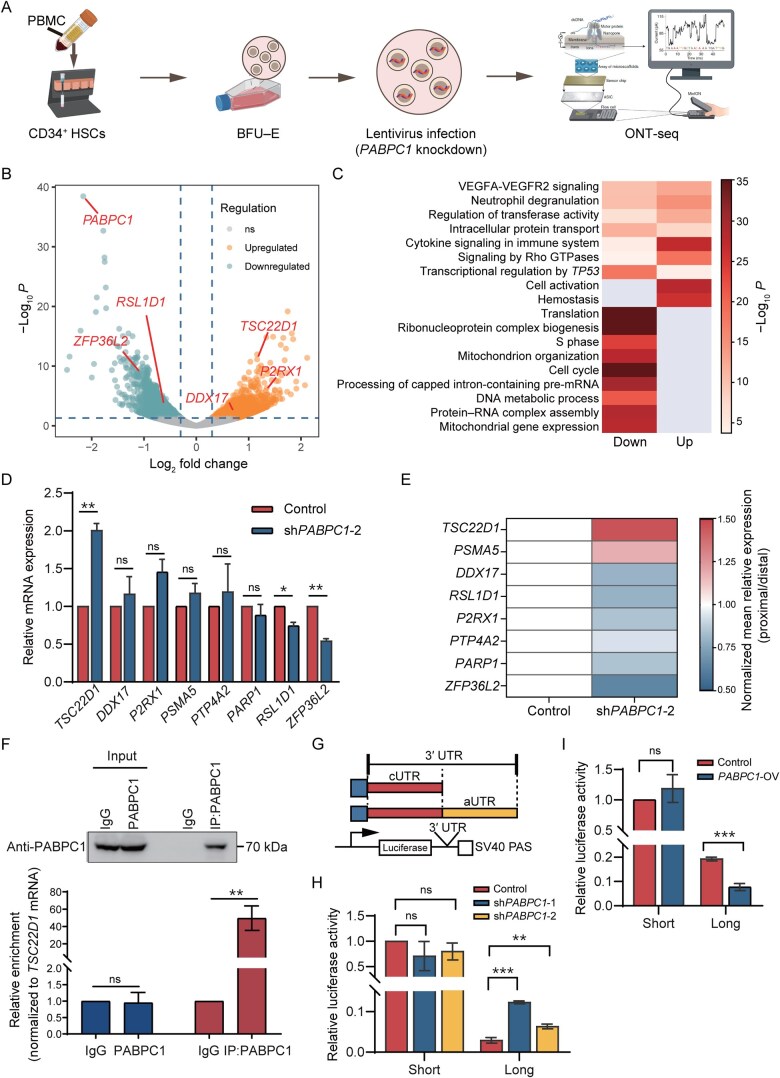
PABPC1 knockdown significantly affects APA events from BFU–E to CFU–E during early erythropoiesis **A**. Flow chart of sample treatment and collection for ONT-seq. CD34^+^ HSCs were purified from human mobilized PBMCs, peripheral blood mononuclear cells. BFU–E cells were sorted from the early erythroid lineage induced by CD34^+^ HSCs, and *PABPC1* was knocked down using lentivirus. The *PABPC1*-knockdown BFU–E cells were subjected to ONT-seq. **B**. Differential gene expression analysis of nanopore sequencing data from early erythrocytes with knockdown of *PABPC1*. Blue circles represent downregulated genes and yellow circles represent upregulated genes. **C**. GO enrichment analysis on differentially expressed genes (*PABPC1* knockdown *vs*. control) in early erythropoiesis. **D**. The validation of mRNA expression of differentially expressed genes by qRT-PCR. *, *P* < 0.05; **, *P* < 0.01; ns, not significant (Student’s *t*-test). **E**. Heatmap representing normalized mean relative expression of the proximal to the distal mRNA isoform based on qRT-PCR analysis (*PABPC1* knockdown *vs*. control). The ratio > 1 indicates a relative increase of the proximal transcript, whereas the ratio < 1 indicates a relative increase of the distal transcript. **F**. Top: validation of antibody specificity by IP**–**Western blotting assay. Lane 1 and Lane 2: input; Lane 3: IgG negative control; Lane 4: IP sample using anti-PABPC1 antibody. Bottom: the RIP assay was performed using normal rabbit IgG or anti-PABPC1 in BFU–E cell lysates. The relative expression level of TSC22D1 was detected by qRT-PCR. ∗∗, *P* < 0.01; ns, not significant (Student's *t-test*). **G**. Schematic of APA isoforms using pPASs or dPASs in the 3′ UTR. The proximal and distal regulatory sequences of the 3′ UTR of the *TSC22D1* gene were cloned into the pGL3-Basic reporter vector, respectively, and validated by sequencing. **H**. Luciferase reporter assays to evaluate the impact of *PABPC1* knockdown on the regulatory efficiency of functional variants in the proximal or distal regions of the 3′ UTR of the *TSC22D1* gene. Luciferase activity was measured using a dual-luciferase detection system. Quantitative analysis was conducted by calculating the Fluc/Rluc ratio. **, *P* < 0.01; ***, *P* < 0.001; ns, not significant (Student’s *t*-test). **I**. Luciferase reporter assays to evaluate the impact of *PABPC1* overexpression on the regulatory efficiency of functional variants in the proximal or distal regions of the 3′ UTR of the *TSC22D1* gene. Luciferase activity was measured using a dual-luciferase detection system. Quantitative analysis was conducted by calculating the Fluc/Rluc ratio. ***, *P* < 0.001; ns, not significant (Student’s *t*-test). HSC, hematopoietic stem cell; PBMC, peripheral blood mononuclear cell; ONT-seq, Oxford Nanopore Technologies direct RNA sequencing; *TSC22D1*, TSC22 domain family member 1; *DDX17*, dead-box helicase 17; *P2RX1*, purinergic receptor P2X 1; *PSMA5*, proteasome subunit alpha type 5; *PTP4A2*, protein tyrosine phosphatase 4A2; *PARP1*, poly(ADP-ribose) polymerase 1; *RSL1D1*, ribosomal L1 domain containing 1; *ZFP36L2*, zinc finger protein 36, C3H type-like 2; GO, Gene Ontology; IP, immunoprecipitation; RIP, RNA immunoprecipitation Fluc, firefly luciferase; Rluc, renilla luciferase.

To explore the transcriptional role of PABPC1 during early erythropoiesis, we knocked down *PABPC1* at the early stage of erythropoiesis and performed nanopore sequencing (**Figure 5**A). Nanopore third-generation sequencing technology generates full-length transcripts without fragmentation or amplification. This method accurately identifies transcript structural features, including alternative splicing (AS), fusion genes, and non-coding RNAs, and provides direct quantification at gene and transcript levels. Additionally, the Oxford Nanopore Technologies direct RNA sequencing (ONT-seq) method detects multiple base modifications directly, thereby reducing sequence bias introduced by PCR [43,44].

We used LAPA for a comprehensive global analysis of APA based on ONT-seq data (see Materials and methods). Our analysis revealed that *PABPC1* knockdown consistently increased pPAS usage across different thresholds (Table S1), suggesting that PABPC1 deficiency shifts PAS usage toward proximal sites. To assess the relationship between poly(A) tail length and APA site selection, we extracted poly(A) tails from all samples and quantified their lengths (Table S2). A paired Wilcoxon signed-rank test demonstrated that distal isoforms consistently possessed significantly longer poly(A) tails than proximal isoforms (median 27.25 nt *vs*. 24.0 nt; *P* = 8.1 × 10^−201^; Figure S4A; Table S3). Moreover, poly(A) tail lengths were significantly reduced in *PABPC1*-depleted samples relative to controls for both distal and proximal isoforms (distal: *P* = 2.1 × 10^−41^; proximal: *P* = 6.6 × 10^−63^; Figure S4B), underscoring its role in poly(A) tail length maintenance. To further dissect the relationship between tail shortening and APA dynamics, we evaluated the distribution of APA switching among genes exhibiting poly(A) tail shortening. Within this cohort, genes undergoing proximal PAS upregulation substantially outnumbered those exhibiting increased distal PAS usage (two-sided binomial test, *P* = 1.86 × 10^−9^; Figure S4C). This suggests that *PABPC1* simultaneously regulates poly(A) tail length and APA site selection; its absence tips the balance toward proximal site usage and shorter poly(A) tails.

On the other hand, our differential gene expression analysis identified 1696 upregulated and 2042 downregulated genes, including significant downregulation of *PABPC1* itself (log_2_ fold-change = −2.16, *P* = 3.45E−39; Figure 5B). Subsequently, Gene Ontology (GO) analysis revealed distinct regulatory trends. Upregulated genes were predominantly linked to cell activation, hemostasis, and cytokine signaling in the immune system. Conversely, downregulated genes were associated with the cell cycle, translation, and ribonucleoprotein complex biogenesis (Figure 5C).

Given the crucial roles of the pathways, such as cell cycle and cellular activation in APA and erythropoiesis, we selected a set of genes differentially expressed in these biological processes to examine their expression and APA changes. Under *PABPC1* knockdown conditions, qRT-PCR analysis indicated increased expression of *TSC22D1*,* DDX17*, and *P2RX1* and decreased expression of *ZFP36L2* and *RSL1D1*, consistent with our RNA-seq results (Figure 5D).

For APA analysis, we quantified the pPASs and dPASs using qRT-PCR and calculated relative pPAS usage. We observed a general increase in dPASs usage for *DDX17*,* P2RX1*, and *ZFP36L2*. In contrast, *TSC22D1* and *PSMA5* showed significantly increased pPAS usage, correlating with elevated expression (Figure 5E). IGV snapshots confirmed increased pPAS usage in *TSC22D1* and APA alterations in other significant genes (Figure S5A–D). To validate the signals at the distal site, we quantified PAS usage by qRT-PCR. *PABPC1* knockdown resulted in a significantly increased pPAS usage and decreased distal site usage (Figure S5E). To comprehensively assess PABPC1’s regulatory role in APA, we employed both knockdown and overexpression methods. RIP assays confirmed an interaction between *TSC22D1* mRNA and PABPC1 protein in erythroid progenitor cells. mRNA enrichment was significantly higher in the PABPC1 group compared to the IgG control (Figure 5F). We constructed firefly luciferase reporters containing either (1) the cUTR of *TSC22D1*, corresponding to pPAS usage, or (2) extended 3′ UTRs incorporating both the cUTR and aUTR regions from the longer APA isoform (Figure 5G). To standardize transcript processing, all constructs were terminated with a heterologous SV40 PAS (Figure 5G). After transfection of equimolar reporter constructs into erythroid progenitor cells, we measured luciferase activity to evaluate APA-mediated regulatory effects. Notably, luciferase expression driven by pPAS-derived 3′ UTRs was stable despite PABPC1 manipulation (Figure 5H and I, Figure S6A and B). In contrast, the extended 3′ UTR displayed significantly reduced luciferase activity upon *PABPC1* overexpression and increased activity upon knockdown (Figure 5H and I, Figure S6A and B). These findings demonstrate that PABPC1 is critical for maintaining dPAS selection in *TSC22D1*, thereby modulating *TSC22D1* expression through the APA mechanism.

### PABPC1 mediates expansion and the transition from BFU–E to CFU–E by influencing *TSC22D1* APA changes

The TSC22D protein family encompasses a range of activities involved in the regulation of cell proliferation and differentiation [45,46]. Among these family members, TSC22D1, the first identified member, is well-known for its tumor suppressor function [47]. For instance, in cervical cancer cells, knockdown of the RBP MEX3D inhibits cell proliferation and colony formation while promoting apoptosis by stabilizing *TSC22D1* mRNA [47].

**Figure 6:**
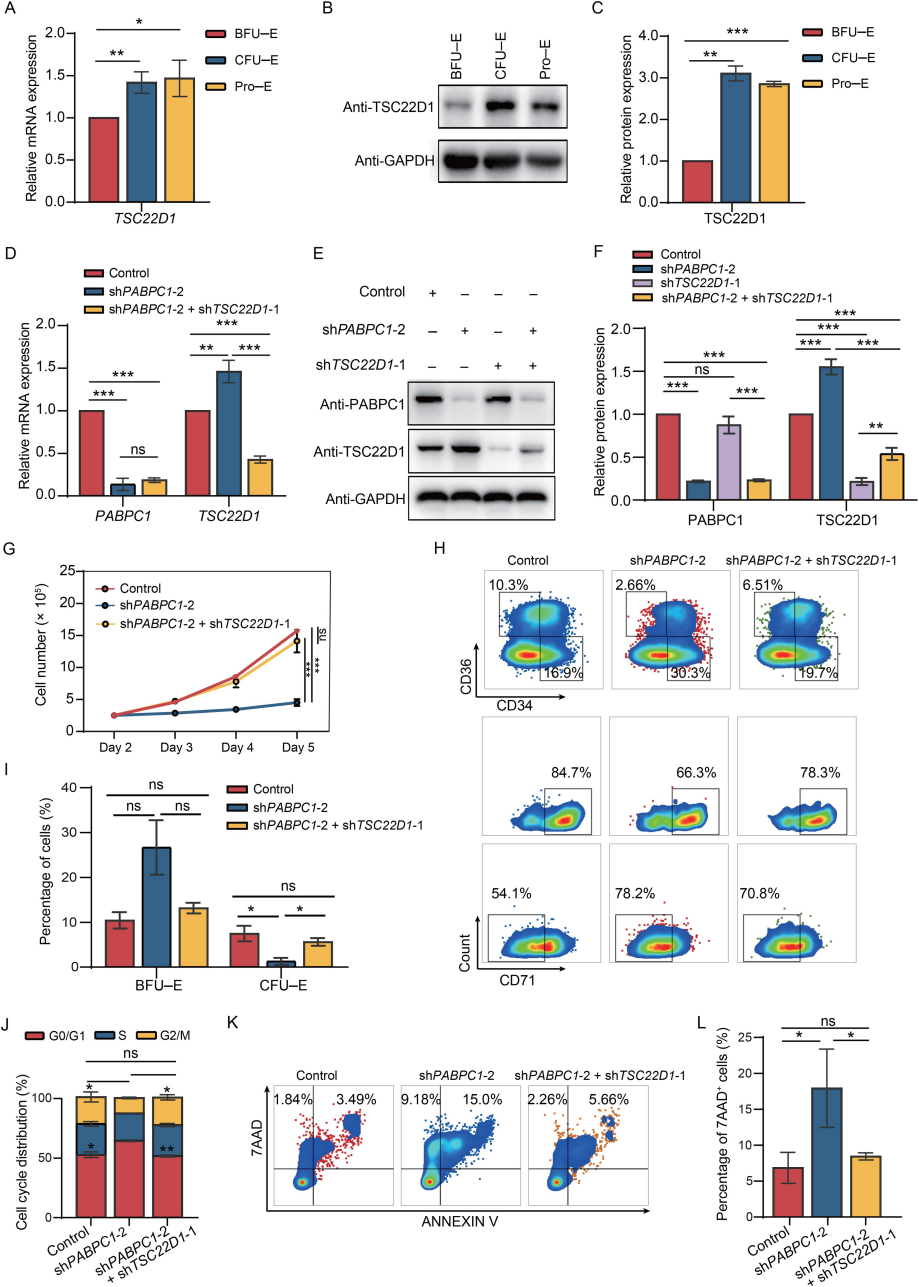
PABPC1 mediates expansion and the transition from BFU–E to CFU–E by influencing ***TSC22D1*** APA changes **A**. qRT-PCR shows the mRNA expression of *TSC22D1* in early erythropoiesis. *, *P* < 0.05; **, *P* < 0.01 (Student’s *t*-test). **B**. Representative images of Western blotting showing TSC22D1 expression in early erythropoiesis. **C**. Quantitative analysis of TSC22D1 expression data from three independent experiments in early erythropoiesis. GAPDH was used as a loading control. **, *P* < 0.01; ***, *P* < 0.001 (Student's *t*-test). **D**. qRT-PCR shows *PABPC1* and *TSC22D1* mRNA expression in early erythroblasts infected with lentivirus containing control shRNA, *PABPC1* shRNA, or *PABPC1* shRNA + *TSC22D1* shRNA. **, *P* < 0.01; ***, *P* < 0.001; ns, not significant (Student’s *t*-test). **E**. Western blotting shows PABPC1 and TSC22D1 expression in early erythroblasts infected with lentivirus containing control shRNA, *PABPC1* shRNA, *TSC22D1* shRNA, or *PABPC1* shRNA + *TSC22D1* shRNA. **F**. Quantitative analysis of PABPC1 and TSC22D1 expression in early erythropoiesis. GAPDH was used as a loading control. **, *P* < 0.01; ***, *P* < 0.001; ns, not significant (Student’s *t*-test). **G**. Early erythroid cell (BFU–E) growth curves were determined by counting cells following infection with the same amount of lentivirus as indicated in (D). Statistical analysis of the data was based on three independent experiments, with the bar plot showing the mean ± SD of triplicate samples. Statistical significance was determined using Student’s *t*-test to compare the knockdown and control groups. ***, *P* < 0.001; ns, not significant. **H**. Representative flow cytometry data of early differentiation for early erythroid cells (BFU–E) infected with the same amount of lentivirus as indicated in (E). CD34^−^CD36^+^CD71^high^ represents the stage of CFU–E cells, while CD34^+^CD36^−^CD71^low^ represents the stage of BFU–E cells. CD34^−^CD36^+^CD71^high^ and CD34^+^CD36^−^CD71^low^ cells were gated from CD45^+^GPA^−^CD123^−^ cells. **I**. Statistical analysis of the percentages of CD34^−^CD36^+^CD71^high^ (CFU–E) and CD34^+^CD36^−^CD71^low^ (BFU–E) cells from three independent experiments is shown. *, *P* < 0.05; ns, not significant (Student’s *t*-test). **J**. Statistical analysis of the PI fluorescence intensity representing each stage of the cell cycle (G0/G1, S, and G2/M) from three independent experiments is shown. *, *P* < 0.05; **, *P* < 0.01; ns, not significant (Student’s *t*-test). **K**. Representative flow cytometry data of cell apoptosis for early erythroid cells (BFU–E) infected with the same amount of lentivirus as indicated in (E). **L**. Statistical analysis of the apoptotic cell rate from three independent experiments is shown. *, *P* < 0.05; ns, not significant (Student’s *t*-test). 7-AAD, 7-aminoactinomycin D.

To determine whether PABPC1 influences the proliferation of early erythroid progenitor cells through TSC22D1 modulation, we examined *TSC22D1* expression from the BFU–E to Pro–E stages. Database analysis and qPCR verification both indicated a significant increase in *TSC22D1* mRNA levels from the BFU–E to CFU–E stages, showing an opposite trend to that observed for *PABPC1* (Figure 3D, **Figure 6**A, Figure S7A). Western blotting assays confirmed these results at the protein level (Figures 3E, 6B, and 6C), suggesting a negative regulation of *TSC22D1* expression by PABPC1.

Subsequently, we conducted sequential knockdowns of *PABPC1* and *TSC22D1*. Knockdown of *PABPC1* increased both *TSC22D1* mRNA and protein expression, which was reversed by subsequent knockdown of *TSC22D1*, without affecting *PABPC1* levels (Figure 6D–F). Further experiments revealed that *PABPC1* knockdown increased *TSC22D1* expression, reduced cell proliferation, hindered differentiation (notably decreasing CFU**–**E cells), increased cell apoptosis, and induced cell cycle arrest at the G0/G1 phase. These effects were rescued by *TSC22D1* knockdown, which restored *TSC22D1* levels following *PABPC1* knockdown (Figure 6G–L). Therefore, these results indicate that PABPC1 regulates the ability of cells to expand and differentiate through APA-mediated changes in *TSC22D1*.

## Discussion

**Figure 7:**
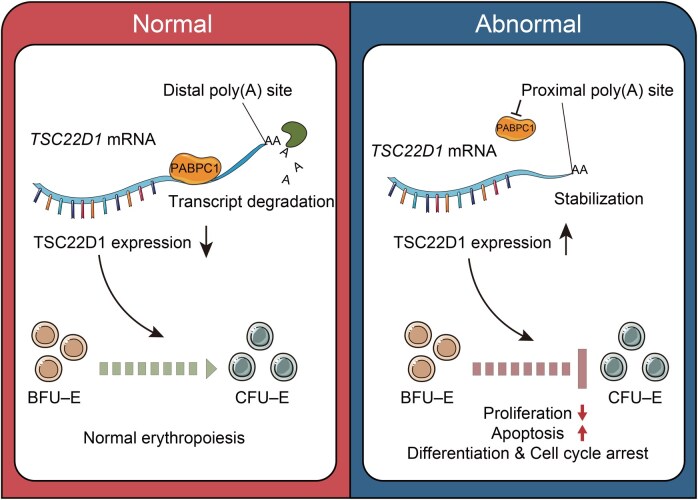
A model of the role of APA in early erythropoiesis PABPC1 controls the expression of *TSC22D1* by regulating *TSC22D1* APA events, thereby modulating the expansion and differentiation of early erythroid progenitor cells.

Our study provides the first comprehensive characterization of alternative polyadenylation (APA) dynamics during erythroid progenitor development. By systematically analyzing APA events across erythroid differentiation stages, we revealed a previously unrecognized wave of APA remodeling during the BFU–E to CFU–E transition. Importantly, we identified PABPC1 as a master regulator of APA in early erythroid progenitors, establishing a functional link between APA regulation and erythroid progenitor expansion. These findings not only advance our understanding of post-transcriptional regulation in erythropoiesis but also provide a molecular framework for investigating progenitor cell homeostasis in both physiological and pathological contexts (**Figure 7**).

Erythropoiesis is tightly regulated to maintain homeostasis, and dysregulation of this process underlies various hematological disorders, including polycythemia vera (PV), myelodysplastic syndrome (MDS), and thalassemia [7–10]. Despite progress in characterizing transcriptional regulators, the post-transcriptional mechanisms governing the erythroid progenitor stage remain poorly understood. Growing evidence implicates APA as a critical regulatory layer in cell fate determination, with particular importance in stem/progenitor cell populations [29,30]. While APA has been reported at the terminal stage of erythroid cells [28], its dynamic regulation during early erythropoiesis — a phase characterized by rapid progenitor expansion — has not yet been systematically investigated.

Our study focuses on the role of APA in erythropoiesis. We applied the DaPars v2.0 algorithm to RNA-seq data from different erythroid developmental stages. We observed significant APA changes during the transition from BFU–E to CFU–E, which are early stages in erythroid progenitor development. This finding indicates that APA is crucial for the growth and self-renewal of erythroid progenitor cells. In addition, by analyzing the proportion of core APA regulatory factors and combining this with gene expression analyses, we identified PABPC1 as a crucial regulator of APA during the BFU–E to CFU–E transition.

PABPC1 is a well-known cytoplasmic poly(A)-binding protein that is expressed in most eukaryotic organisms [39]. Previous studies have reported that knocking down PABPC1 can inhibit the proliferation and progression of cancer cells [48–50]. This factor plays a dual role by protecting mRNA poly(A) and promoting deadenylation, demonstrating a complex regulatory mechanism, and is involved in maintaining transcripts with long 3′ UTR isoforms [39,41]. Previous research has documented that knockdown of *PABPC1* leads to 3′ UTR shortening through APA events and inhibits the progression and metastasis of various cancers [39,50]. Our results further demonstrate that PABPC1 maintains transcript stability with long 3′ UTR isoforms. Notably, the erythroid-specific consequences of *PABPC1* depletion impaired proliferation, differentiation arrest, and altered survival, suggesting its regulatory outputs are context-dependent and finely tuned to hematopoietic demands. This aligns with emerging paradigms of APA regulation as a cell type-specific mechanism for coordinating proliferation and differentiation.

Our mechanistic analysis revealed TSC22D1 as a critical effector linking PABPC1-mediated APA regulation to erythroid progenitor expansion. The observed 3′ UTR shortening and consequent overexpression of *TSC22D1* upon *PABPC1* knockdown provide direct experimental evidence for APA-mediated gene regulation in erythropoiesis. Given that TSC22D1 functions downstream of TGF-β signaling — a pathway known to constrain BFU–E expansion [45,51], our findings suggest an elegant regulatory circuit: PABPC1 maintains physiological *TSC22D1* mRNA expression levels via 3′ UTR-mediated control, thereby fine-tuning TGF-β signaling output to permit progenitor proliferation. This mechanistic model integrates post-transcriptional regulation with established signaling pathways, offering new therapeutic entry points for manipulating erythropoietic output.

Although our results suggest that APA plays a role in erythropoiesis, this study does not directly link APA disruptions to specific erythropoiesis disorders, such as PV, MDS, and thalassemia. Additionally, PABPC1 is just one of several core APA factors involved. Other APA regulatory proteins and their potential interactions with PABPC1 have yet to be fully explored. Thus, further studies are essential to map out the complete landscape of APA regulation and its implications for erythroid-related disease pathology.

In conclusion, our study represents a significant advance in the understanding of APA regulation during erythropoiesis, particularly highlighting the pivotal role of PABPC1. By demonstrating the impact of APA modulation on important genes like *TSC22D1*, we provide new insights into the molecular mechanisms governing erythropoiesis. Our findings establish APA regulation as a previously unrecognized pillar of the control of erythroid development, opening new avenues for investigating both normal and pathological erythropoiesis. Future studies exploring APA dynamics in primary patient samples and genetically engineered models will be crucial for translating these discoveries into clinical insights.

## Materials and methods

### APA analysis

To explore the APA pattern during erythroid differentiation, we collected RNA-seq data for human early erythropoiesis (BFU–E to CFU–E stages) from GSE61566, and RNA-seq data for human erythroid terminal differentiation (Pro–E to Ortho–E stages) from GSE53983. Each stage in these datasets contains 3 samples across 7 erythroid stages for a total of 21 samples. We then converted RNA-seq file from SRA format to fastq format using fastq-dump, and then aligned the sequence to human genome (hg19) by HISAT2. The 21 generated BAM files were then utilized as input for DaPars v2.0, which employs a two-normal mixture model to calculate the percentage of PDUI, a metric for assessing APA usage for each gene (defined as APA event). PDUI values range from 0 to 1, where higher values indicate increased utilization of dPASs. To ensure the precision of our predictions, we set a coverage threshold for the last exon of ≥ 30×, following the recommendations of DaPars (v2.0). To investigate 3′ UTR lengthened/shortened genes during erythropoiesis, we compared PDUI values between different stages and set |ΔPDUI| ≥ 0.15 as the criterion. If PDUI at one stage is higher than that of the previous stage, the 3′ UTR of the APA event is lengthening during differentiation, and *vice versa*.

To investigate whether PABPC1 regulates APA, we analyzed third-generation sequencing data derived from erythroid progenitor cells, including control and *PABPC1* knockdown samples. We employed LAPA (https://github.com/mortazavilab/lapa/), a tool specifically designed for third-generation sequencing APA analysis, to systematically assess PASs usage in both the *PABPC1* knockdown and control samples. Specifically, we selected the two sites exhibiting the greatest variation in usage as the pPASs and dPASs. PASs choice was quantified based on the following criteria: (1) pPASs usage choice: an increase in proximal site usage accompanied by a decrease in distal site usage. (2) dPASs choice: an increase in distal site usage accompanied by a decrease in proximal site usage.

Finally, we applied a range of thresholds (±0, ±0.1, ±0.15, and ±0.2) as the criteria to determine poly(A) usage changes.

### Analysis of poly(A) signal motif

A series of *cis*-elements that regulate APA are enriched near the poly(A) sites. To identify potential *cis*-elements that affect APA during erythroid development, BEDtools was used to extract the genome sequences ±50 bp around pPASs and dPASs, respectively. After obtaining the sequences, DREME was used to enrich the features to detect poly(A) signal motifs of pPASs and dPASs.

### Prediction of upstream 3′ end processing factors regulating APA

To screen for upstream 3′ end processing factors regulating APA during erythroid differentiation, we collected a list of reported APA core factors from https://www.nature.com/articles/ncomms6274. Pearson’s correlation analysis was employed to assess the relationship between the PDUI values of each APA event and the expression levels of corresponding APA core factors. The criteria for determining the significance were stringently defined as FDR ≤ 0.05 and *r*^2^ ≥ 0.3. Finally, the proportion of APA events linked to APA core factors was counted, and APA core factors that might affect erythroid differentiation were further screened according to the number of APA events with significant differences.

### Poly(A) tail length analysis

To extract poly(A) tail length information, we employed a script available at https://github.com/ankeetashah/Benchmarking-APA/blob/main/scripts/01_filter_for_polyA.py, which extracts read IDs, poly(A) tail lengths, and transcript lengths from our ONT-seq files. After extracting the genomic positions of these read IDs from the BAM files, we matched these positions with APA-related poly(A) site peaks identified by LAPA, thereby obtaining poly(A) tail length information for APA genes. Based on our previous LAPA analysis, each APA gene retained two major peaks — defined as distal and proximal sites — and we compared the median poly(A) tail lengths between these sites within the same gene to determine which had longer tails.

### Cell source and culture

Human mobilized peripheral blood samples were obtained from Xiangya Hospital and the Second Xiangya Hospital of Central South University, China. CD34^+^ cells were purified from human mobilized peripheral blood. The isolated CD34^+^ cells were maintained in a two-stage liquid culture system. During the initial phase (day 0 to day 6), 1 × 10^5^/ml CD34^+^ cells were seeded (day 0) in Serum-Free Expansion Medium containing 10% FBS, 1 U/ml EPO (Catalog No. 78007, Stem Cell Technology, Vancouver, Canada), 10 ng/ml IL-3 (Catalog No. 78040, Stem Cell Technology), 50 ng/ml SCF (Catalog No. 78062, Stem Cell Technology) and 0.06 mM α-thioglycerol (Catalog No. M6145, Sigma, Louis, MO). On day 4, fresh medium was added to dilute the cells, and incubation proceeded until day 7. In the subsequent phase (day 7 to day 13), cells were cultured at a density of 1 × 10^5^ cells/ml in SFEM medium supplemented with 30% FBS, along with 1 U/ml EPO and α-thioglycerol [3].

### Lentivirus infection

*PABPC1* shRNAs and control shRNA were obtained from GenePharma Co., Ltd. (Shanghai, China). shRNA targeting sequences: shControl, 5′-TTCTCCGAACGTGTCACGT-3′; sh*PABPC1*-1, 5′-GGACAAATCCATTGATAAT-3′; sh*PABPC1*-2, 5′-GAAAGGAGCTCAATGGAAA; sh*TSC22D1*-1, 5′-GAGTTTACCAACTGAGACATT-3′; sh*TSC22D1*-2, 5′-GCAAGCTATGGATCTAGTGAA-3′. Thirty million lentiviruses were used to infect 0.5 million CD34^+^ cells on day 2, and 12 h after infection, cells were washed with PBS and cultured in new complete medium.

### RNA extraction and qRT-PCR analysis

RNA extracted from primary cultured human erythroid cells using TRIZOL Reagent (Catalog No. R401-01, Vazyme, Nanjing, China) was reverse-transcribed with HiScript II Q RT SuperMix for qPCR (+gDNA wiper) Kit (Catalog No. R223-01, Vazyme, China) according to the manufacturer’s guidelines, and amplified by quantitative polymerase chain reaction (qRT-PCR) with the Cycler and appropriate primer pairs. The primers used for qRT-PCR are summarized in Table S4. Relative gene expression was calculated using the 2^−^^ΔΔCT^ method, with *GAPDH* used as the reference gene. For analysis of APA switches by qRT-PCR, the 2^−^^ΔCT^ value was calculated. The proximal 2^−^^ΔCT^ value was divided by the distal 2^−^^ΔCT^ value, and the results were normalized. Results > 1 indicate a relative increase of the proximal transcript, whereas results < 1 indicate a relative increase of the distal transcript [28]. Primers were designed using NCBI Primer-BLAST.

### Western blotting analysis

For Western blotting analysis, whole-cell lysates from cultured cells were prepared with RIPA buffer in the presence of cocktail of protease inhibitor (Catalog No. 05892791001, Roche, Basel, Switzerland) and PhosSTOP phosphatase inhibitor (Catalog No. 04906837001). Protein concentration was measured using a Pierce BCA protein assay kit (Catalog No. 23227, Thermo Fisher Scientific, Waltham, MA). Protein extracts (30 μg) were boiled, subjected to 10% SDS-PAGE, and then transferred to nitrocellulose membranes. The membranes were blocked with 5% nonfat dry milk and incubated with specific primary antibodies (diluted 1:2000) at 4°C overnight. The HRP-conjugated secondary antibodies were used at 1:3000 dilutions for 1.5 h at room temperature. Signals were detected using ECL HRP substrate (Catalog No. P0018AM, Beyotime Shanghai, China). Western blotting analysis was performed as described previously.

### RNA immunoprecipitation assay

RNA immunoprecipitation (RIP) was performed using a Magna RBP immunoprecipitation kit (Catalog No.17-700, Millipore, Bedford, MA) according to the manufacturer’s instructions. Briefly, cells were collected and lysed in complete RIPA buffer containing a protease inhibitor cocktail and RNase inhibitor. Next, the cell lysates were incubated with RIP buffer containing magnetic beads conjugated with human anti-PABPC1 antibody or control normal rabbit IgG. The samples were digested with proteinase K to isolate the immunoprecipitated RNA. The purified RNA was finally subjected to real-time PCR to demonstrate the presence of the binding targets.

### Plasmid construction and dual-luciferase reporter assay

We employed the luciferase reporter system to evaluate the impact of PABPC1 knockdown or overexpression on the regulatory efficiency of functional variants in the proximal or distal regions of the *TSC22D1* gene 3′ UTR. The experimental strategy was as follows: First, the proximal and distal regulatory sequences of the *TSC22D1* gene 3′ UTR were cloned into the PGL3-BASIC reporter vector and validated by sequencing. Subsequently, functional validation was performed using a dual-luciferase reporter system: 293T cells seeded in 12-well plates were subjected to lentiviral infection to establish stable *TSC22D1* knockdown or overexpression cell models. Following confirmation of gene expression modulation by Western blotting, the constructed reporter plasmids and pRL-TK internal control plasmid were co-transfected using Lipofectamine 2000 transfection reagent (Catalog No. 11668019, Invitrogen, Carlsbad, CA). Cells were harvested 48 h post-transfection, and luciferase activity was measured using a dual-luciferase detection system. Quantitative analysis was conducted by calculating the Fluc/Rluc ratio.

### Cell proliferation experiment

After infection with lentivirus, 50,000 cells were taken from each group and placed in a 24-well plate for culturing. On days 2, 3, 4, and 5, the cells were gently blown and counted.

### Flow-cytometric analysis

For apoptosis analysis, 5 × 10^5^ cells were collected, washed with PBS, and resuspended in 100 μl 1× Annexin V Binding buffer (Catalog No. FL0100, Zeta Life, San Francisco, CA). Cells were then stained with 5 μl Annexin V-FITC and 5 μl PI according to the manufacturer’s protocol (Catalog No. FL0100, Zeta Life). After incubating at room temperature for 15 min in the dark, cells were subjected to flow-cytometric analysis on a FACS LSRII flow cytometer (BD Biosciences, Franklin Lakes, NJ). For analysis of early erythroblast differentiation, cells were collected at 48 h after lentivirus infection. A total of 2 × 10^5^ cells were washed and resuspended. Cells were then stained with CD45-APC-Cy7, GPA-BV605, CD36-FITC, CD34-APC, CD71-PE, and CD123-PE-Cy7 antibodies at 4°C for 20 min in the dark. Samples were washed once with PBS and stained with 7AAD-PerCP-Cy5.5 before analysis, then subjected to flow-cytometric analysis on a LSR II Flow Cytometer (BD Biosciences). Unstained cells and APC-Cy7-, BV605-, FITC-, APC-, PE-, PE Cy7-, and PerCP-Cy5.5-stained cells were used as negative controls. All flow-cytometric data were analyzed with FlowJo 10.4.

### Colony experiment

Cultured primary CD34^+^ cells and infected with lentivirus at day 1 were plated in triplicate at a density of 300 cells in 1 ml of MethoCult H4434 classic medium (complete medium containing SCF, IL-3, Epo and GM**–**CSF) (Catalog No. 04100, StemCell, Vancouver, BC) or in 1 ml of MethoCult H4330 medium (Catalog No. 04100, StemCell) with Epo only. The CFU–E and BFU–E colonies were defined according to the criteria described by Dover and colleagues [53]. CFU–E colonies were counted on day 7, and BFU–E colonies were counted on day 15.

### Nanopore sequencing

We performed ONT-seq on early erythroid cells treated with *PABPC1* lentiviral knockdown.

## Ethical statement

Human mobilized peripheral blood samples were obtained from Xiangya Hospital and the Second Xiangya Hospital of Central South University with approval from the Ethics Committees (Approval No. 202103711). Informed consent was obtained from all participating subjects. The study was registered with and approved by the Human Genetics Resource (HCR) office of the Minister of Science and Technology of China [Approval No. (2022) BC0004].

## Code availability

The code of this study is available at GitHub (https://github.com/boiscat/Erythrocyte-APA). The code has also been submitted to BioCode at the National Genomics Data Center (NGDC), China National Center for Bioinformation (CNCB) (BioCode: BT007889), which is publicly accessible at https://ngdc.cncb.ac.cn/biocode/tools/BT007889.

## Supplementary Material

qzaf116_Supplementary_Data

## Data Availability

The ONT-seq data have been deposited in the National Center for Biotechnology Information (BioProject: PRJNA1061347), and are publicly accessible at https://www.ncbi.nlm.nih.gov/. The data have also been deposited in the Genome Sequence Archive for Human [52] at NGDC, CNCB (GSA-Human: HRA010761), and are publicly accessible at https://ngdc.cncb.ac.cn/gsa-human.

## CRediT author statement

**Yanan Li:** Investigation, Data curation, Formal analysis, Visualization, Writing – original draft, Writing – review & editing. **Yanbo Yang:** Formal analysis, Data curation, Software, Visualization, Writing – original draft, Writing – review & editing. **Bin Hu:** Investigation, Data curation, Validation. **Zi Wang:** Investigation, Data curation. **Wei Wang:** Resources. **Xiaofeng He:** Resources. **Xusheng Wu:** Resources. **Sheng Lin:** Resources. **Narla Mohandas:** Writing – review & editing. **Hong Liu:** Conceptualization, Project administration, Funding acquisition. **Jing Gong:** Conceptualization, Project administration, Funding acquisition. **Long Liang:** Conceptualization, Project administration, Funding acquisition, Writing – original draft, Writing – review & editing. **Jing Liu:** Conceptualization, Project administration, Funding acquisition, Supervision, Writing – review & editing. All authors have read and approved the final manuscript.

## References

[1] Yamamoto R , Morita Y, Ooehara J, Hamanaka S, Onodera M, Rudolph KL, et al Clonal analysis unveils self-renewing lineage-restricted progenitors generated directly from hematopoietic stem cells. Cell 2013;154:1112–26. 23993099 10.1016/j.cell.2013.08.007

[2] Dong F , Hao S, Zhang S, Zhu C, Cheng H, Yang Z, et al Differentiation of transplanted haematopoietic stem cells tracked by single-cell transcriptomic analysis. Nat Cell Biol 2020;22:630–9. 32367048 10.1038/s41556-020-0512-1

[3] Li J , Hale J, Bhagia P, Xue F, Chen L, Jaffray J, et al Isolation and transcriptome analyses of human erythroid progenitors: BFU-E and CFU-E. Blood 2014;124:3636–45. 25339359 10.1182/blood-2014-07-588806 PMC4256913

[4] Li Y , Zhang H, Hu B, Wang P, Wang W, Liu J. Post-transcriptional regulation of erythropoiesis. Blood Sci 2023;5:150–9. 37546708 10.1097/BS9.0000000000000159 PMC10400058

[5] Han X , Zhang J, Peng Y, Peng M, Chen X, Chen H, et al Unexpected role for p19INK4d in posttranscriptional regulation of GATA1 and modulation of human terminal erythropoiesis. Blood 2017;129:226–37. 27879259 10.1182/blood-2016-09-739268 PMC5234221

[6] Peng Y , Tang L, Li Y, Song J, Liu H, Wang P, et al Comprehensive proteomic analysis reveals dynamic phospho-profiling in human early erythropoiesis. Br J Haematol 2022;199:427–42. 35974424 10.1111/bjh.18407

[7] Iskander D , Psaila B, Gerrard G, Chaidos A, Foong HE, Harrington Y, et al Elucidation of the EP defect in Diamond-Blackfan anemia by characterization and prospective isolation of human EPs. Blood 2015;125:2553–7. 25755292 10.1182/blood-2014-10-608042

[8] Nandakumar SK , Ulirsch JC, Sankaran VG. Advances in understanding erythropoiesis: evolving perspectives. Br J Haematol 2016;173:206–18. 26846448 10.1111/bjh.13938 PMC4833665

[9] Hellström-Lindberg E , Tobiasson M, Greenberg P. Myelodysplastic syndromes: moving towards personalized management. Haematologica 2020;105:1765–79. 32439724 10.3324/haematol.2020.248955 PMC7327628

[10] Cazzola M. Ineffective erythropoiesis and its treatment. Blood 2022;139:2460–70. 34932791 10.1182/blood.2021011045

[11] Gao X , Lee HY, da Rocha EL, Zhang C, Lu YF, Li D, et al TGF-β inhibitors stimulate red blood cell production by enhancing self-renewal of BFU-E erythroid progenitors. Blood 2016;128:2637–41. 27777239 10.1182/blood-2016-05-718320 PMC5146747

[12] Zhang L , Prak L, Rayon-Estrada V, Thiru P, Flygare J, Lim B, et al ZFP36L2 is required for self-renewal of early burst-forming unit erythroid progenitors. Nature 2013;499:92–6. 23748442 10.1038/nature12215 PMC3702661

[13] Gruber AJ , Zavolan M. Alternative cleavage and polyadenylation in health and disease. Nat Rev Genet 2019;20:599–614. 31267064 10.1038/s41576-019-0145-z

[14] Mitschka S , Mayr C. Context-specific regulation and function of mRNA alternative polyadenylation. Nat Rev Mol Cell Biol 2022;23:779–96. 35798852 10.1038/s41580-022-00507-5 PMC9261900

[15] Tian B , Manley JL. Alternative polyadenylation of mRNA precursors. Nat Rev Mol Cell Biol 2017;18:18–30. 27677860 10.1038/nrm.2016.116 PMC5483950

[16] Zhang Y , Liu L, Qiu Q, Zhou Q, Ding J, Lu Y, et al Alternative polyadenylation: methods, mechanism, function, and role in cancer. J Exp Clin Cancer Res 2021;40:51. 33526057 10.1186/s13046-021-01852-7 PMC7852185

[17] Brumbaugh J , Di Stefano B, Wang X, Borkent M, Forouzmand E, Clowers KJ, et al Nudt21 controls cell fate by connecting alternative polyadenylation to chromatin signaling. Cell 2018;172:629–31. 29373832 10.1016/j.cell.2017.12.035 PMC5831378

[18] Qin H , Ni H, Liu Y, Yuan Y, Xi T, Li X, et al RNA-binding proteins in tumor progression. J Hematol Oncol 2020;13:90. 32653017 10.1186/s13045-020-00927-w PMC7353687

[19] Blake D , Lynch KW. The three as: alternative splicing, alternative polyadenylation and their impact on apoptosis in immune function. Immunol Rev 2021;304:30–50. 34368964 10.1111/imr.13018 PMC8616797

[20] Witkowski MT , Lee S, Wang E, Lee AK, Talbot A, Ma C, et al NUDT21 limits CD19 levels through alternative mRNA polyadenylation in B cell acute lymphoblastic leukemia. Nat Immunol 2022;23:1424–32. 36138187 10.1038/s41590-022-01314-y PMC9611506

[21] Xia Z , Donehower LA, Cooper TA, Neilson JR, Wheeler DA, Wagner EJ, et al Dynamic analyses of alternative polyadenylation from RNA-seq reveal a 3′-UTR landscape across seven tumour types. Nat Commun 2014;5:5274. 25409906 10.1038/ncomms6274 PMC4467577

[22] Lee SH , Singh I, Tisdale S, Abdel-Wahab O, Leslie CS, Mayr C. Widespread intronic polyadenylation inactivates tumour suppressor genes in leukaemia. Nature 2018;561:127–31. 30150773 10.1038/s41586-018-0465-8 PMC6527314

[23] Xiang Y , Ye Y, Lou Y, Yang Y, Cai C, Zhang Z, et al Comprehensive characterization of alternative polyadenylation in human cancer. J Natl Cancer Inst 2018;110:379–89. 29106591 10.1093/jnci/djx223 PMC6059203

[24] Chen Y , Chen B, Li J, Li H, Wang G, Cai X, et al Alternative mRNA polyadenylation regulates macrophage hyperactivation via the autophagy pathway. Cell Mol Immunol 2024;21:1522–34. 39537902 10.1038/s41423-024-01237-8 PMC11607066

[25] Wang L , Lang GT, Xue MZ, Yang L, Chen L, Yao L, et al Dissecting the heterogeneity of the alternative polyadenylation profiles in triple-negative breast cancers. Theranostics 2020;10:10531–47. 32929364 10.7150/thno.40944 PMC7482814

[26] Miles WO , Lembo A, Volorio A, Brachtel E, Tian B, Sgroi D, et al Alternative polyadenylation in triple-negative breast tumors allows NRAS and c-JUN to bypass PUMILIO posttranscriptional regulation. Cancer Res 2016;76:7231–41. 27758885 10.1158/0008-5472.CAN-16-0844 PMC5553310

[27] Song J , Nabeel-Shah S, Pu S, Lee H, Braunschweig U, Ni Z, et al Regulation of alternative polyadenylation by the C2H2-zinc-finger protein Sp1. Mol Cell 2022;82:3135–50.e9. 35914531 10.1016/j.molcel.2022.06.031

[28] Sommerkamp P , Altamura S, Renders S, Narr A, Ladel L, Zeisberger P, et al Differential alternative polyadenylation landscapes mediate hematopoietic stem cell activation and regulate glutamine metabolism. Cell Stem Cell 2020;26:722–38.e7. 32229311 10.1016/j.stem.2020.03.003

[29] Kini HK , Kong J, Liebhaber SA. Cytoplasmic poly(A) binding protein C4 serves a critical role in erythroid differentiation. Mol Cell Biol 2014;34:1300–9. 24469397 10.1128/MCB.01683-13 PMC3993565

[30] van Zalen S , Lombardi AA, Jeschke GR, Hexner EO, Russell JE. AUF-1 and YB-1 independently regulate β-globin mRNA in developing erythroid cells through interactions with poly(A)-binding protein. Mech Dev 2015;136:40–52. 25720531 10.1016/j.mod.2015.02.003 PMC4390516

[31] Feng X , Li L, Wagner EJ, Li W. TC3A: The Cancer 3′ UTR Atlas. Nucleic Acids Res 2018;46:D1027–30. 30053266 10.1093/nar/gkx892 PMC5753254

[32] Ielasi FS , Ternifi S, Fontaine E, Iuso D, Couté Y, Palencia A. Human histone pre-mRNA assembles histone or canonical mRNA-processing complexes by overlapping 3′-end sequence elements. Nucleic Acids Res 2022;50:12425–43. 36447390 10.1093/nar/gkac878 PMC9756948

[33] Liu X , Hoque M, Larochelle M, Lemay JF, Yurko N, Manley JL, et al Comparative analysis of alternative polyadenylation in *S*. *cerevisiae* and *S. pombe*. Genome Res 2017;27:1685–95. 28916539 10.1101/gr.222331.117 PMC5630032

[34] Beaudoing E , Freier S, Wyatt JR, Claverie JM, Gautheret D. Patterns of variant polyadenylation signal usage in human genes. Genome Res 2000;10:1001–10. 10899149 10.1101/gr.10.7.1001 PMC310884

[35] Kwon B , Fansler MM, Patel ND, Lee J, Ma W, Mayr C. Enhancers regulate 3′ end processing activity to control expression of alternative 3′ UTR isoforms. Nat Commun 2022;13:2709. 35581194 10.1038/s41467-022-30525-y PMC9114392

[36] Zhang Q , Tian B. The emerging theme of 3′ UTR mRNA isoform regulation in reprogramming of cell metabolism. Biochem Soc Trans 2023;51:1111–9. 37171086 10.1042/BST20221128 PMC10771799

[37] Agarwal V , Lopez-Darwin S, Kelley DR, Shendure J. The landscape of alternative polyadenylation in single cells of the developing mouse embryo. Nat Commun 2021;12:5101. 34429411 10.1038/s41467-021-25388-8 PMC8385098

[38] Li W , You B, Hoque M, Zheng D, Luo W, Ji Z, et al Systematic profiling of poly(A)+ transcripts modulated by core 3′ end processing and splicing factors reveals regulatory rules of alternative cleavage and polyadenylation. PLoS Genet 2015;11:e1005166. 25906188 10.1371/journal.pgen.1005166 PMC4407891

[39] Zhang Y , Chen C, Liu Z, Guo H, Lu W, Hu W, et al PABPC1-induced stabilization of IFI27 mRNA promotes angiogenesis and malignant progression in esophageal squamous cell carcinoma through exosomal miRNA-21-5p. J Exp Clin Cancer Res 2022;41:111. 35346324 10.1186/s13046-022-02339-9 PMC8962095

[40] Gray NK , Hrabálková L, Scanlon JP, Smith RW. Poly(A)-binding proteins and mRNA localization: who rules the roost? Biochem Soc Trans 2015;43:1277–84. 26614673 10.1042/BST20150171

[41] Kühn U, Gündel M , Knoth A, Kerwitz Y, Rüdel S, Wahle E. Poly(A) tail length is controlled by the nuclear poly(A)-binding protein regulating the interaction between poly(A) polymerase and the cleavage and polyadenylation specificity factor. J Biol Chem 2009;284:22803–14. 19509282 10.1074/jbc.M109.018226 PMC2755688

[42] Apponi LH , Leung SW, Williams KR, Valentini SR, Corbett AH, Pavlath GK. Loss of nuclear poly(A)-binding protein 1 causes defects in myogenesis and mRNA biogenesis. Hum Mol Genet 2010;19:1058–65. 20035013 10.1093/hmg/ddp569 PMC2830829

[43] Deamer D , Akeson M, Branton D. Three decades of nanopore sequencing. Nat Biotechnol 2016;34:518–24. 27153285 10.1038/nbt.3423 PMC6733523

[44] Wang Y , Zhao Y, Bollas A, Wang Y, Au KF. Nanopore sequencing technology, bioinformatics and applications. Nat Biotechnol 2021;39:1348–65. 34750572 10.1038/s41587-021-01108-x PMC8988251

[45] Dragotto J , Canterini S, Del Porto P, Bevilacqua A, Fiorenza MT. The interplay between TGF-β-stimulated TSC22 domain family proteins regulates cell-cycle dynamics in medulloblastoma cells. J Cell Physiol 2019;234:18349–60. 30912127 10.1002/jcp.28468

[46] Canterini S , Carletti V, Nusca S, Mangia F, Fiorenza MT. Multiple TSC22D4 iso-/phospho-glycoforms display idiosyncratic subcellular localizations and interacting protein partners. FEBS J 2013;280:1320–9. 23305244 10.1111/febs.12123

[47] Zheng Z , Chen X, Cai X, Lin H, Xu J, Cheng X. RNA-binding protein MEX3D promotes cervical carcinoma tumorigenesis by destabilizing TSC22D1 mRNA. Cell Death Discov 2022;8:250. 35513372 10.1038/s41420-022-01049-7 PMC9072549

[48] Li J , Pei M, Xiao W, Liu X, Hong L, Yu Z, et al The HOXD9-mediated PAXIP1-AS1 regulates gastric cancer progression through PABPC1/PAK1 modulation. Cell Death Dis 2023;14:341. 37225681 10.1038/s41419-023-05862-5 PMC10209196

[49] Zhu Y , Qi M. Expression and prognostic roles of PABPC1 in hepatocellular carcinoma. Int J Surg 2020;84:3–12. 33080414 10.1016/j.ijsu.2020.10.004

[50] Qi Y , Wang M, Jiang Q. PABPC1–mRNA stability, protein translation and tumorigenesis. Front Oncol 2022;12:1025291. 36531055 10.3389/fonc.2022.1025291 PMC9753129

[51] Lu Y , Kitaura J, Oki T, Komeno Y, Ozaki K, Kiyono M, et al Identification of TSC-22 as a potential tumor suppressor that is upregulated by Flt3-D835V but not Flt3-ITD. Leukemia 2007;21:2246–57. 17690703 10.1038/sj.leu.2404883

[52] Zhang S , Chen X, Jin E, Wang A, Chen T, Zhang X, et al The GSA family in 2025: a broadened sharing platform for multi-omics and multimodal data. Genomics Proteomics Bioinformatics 2025;23:qzaf072. 40857552 10.1093/gpbjnl/qzaf072 PMC12451262

[53] Dover GJ, Chan T, Sieber F. Fetal hemoglobin production in cultures of primitive and mature human erythroid progenitors: differentiation affects the quantity of fetal hemoglobin produced per fetal-hemoglobin-containing cell. Blood 1983;61:1242–6. 6188507

