# Supplementary material for: Regulation of Alternative Polyadenylation Events by PABPC1 Affects Erythroid Progenitor Cell Expansion
Source: Genomics Proteomics Bioinformatics. 2025 Nov 25;23(6):qzaf116. doi: 10.1093/gpbjnl/qzaf116 (PMC13245397; doi:10.1093/gpbjnl/qzaf116)
Supplement: qzaf116_Supplementary_Data [file qzaf116_supplementary_data.zip › Supplementary material captions.docx]

**Supplementary material**

**File S1 The proximal or distal regions of *TSC22D1* gene 3' UTR**

**Figure S1 A comprehensive view of 3' UTR length and clustering characteristics of APA events during erythroid differentiation**

**A.** Boxplot of 3' UTR length variations in transcripts with and without APA events. **B.** Scatter plot showing 3′ UTR size (x-axis) versus aUTR size (y-axis) for each APA event, with Pearson correlation coefficient (r) and *P* value indicated. **C.** Scatter plot showing 3' UTR size (x-axis) versus cUTR size (y-axis) for each APA event, with Pearson correlation coefficient (r) and *P* value indicated.

**Figure S2 Overexpression of *PABPC1* promotes differentiation and expansion of erythroid progenitor cells**

**A.** Representative flow cytometry chart of early differentiation for early erythroid cells (BFU-E) infected with the same amount of *PABPC1* overexpression or control lentivirus. CD34^-^ CD36^+^ CD71^high^ cells represent the stage of CFU**-**E cells, while CD34^+^ CD36^-^ CD71^low^ cells represent the stage of BFU**-**E cells. CD34^-^ CD36^+^ CD71^high^ and CD34^+^ CD36^-^ CD71^low^ cells were gate from CD45^+^ GPA^-^ CD123^-^ cells. **B.** Statistical analysis of the percentage (%) of CD34^-^ CD36^+^ CD71 ^high^ (CFU**–**E) and CD34^+^ CD36^-^ CD71 ^low^ (BFU**-**E) cells from three independent experiments is shown. **C.** Representative images showing the formation of BFU**-**E and CFU**-**E colonies (left panel). Statistical analysis of the number of BFU**-**E and CFU**-**E colonies formed in the colony formation assay (right panel). Data shown are mean ± SD (*n* = 3) (*, *P*  < 0.05; **, *P*  < 0.01; ***, *P*  < 0.001).

**Figure S3 *PABPC4* does not affect the differentiation and expansion of erythroid progenitor cells**

**A.** RT-qPCR analysis of genes mRNA expression in human BFU**-**E, CFU**-**E, and Pro-E populations which sorted by flow cytometry from induced erythroid lineage *in vitro*. The mRNA expression levels in each group were normalized to GAPDH expression. **B.** RT-qPCR analysis of *PABPC4* mRNA expression in early erythroid cells (BFU**-**E) after infection with control lentivirus or *PABPC4* knockdown lentivirus. *GAPDH* was used as an internal control. **C.** Early erythroid cell (BFU**-**E) growth curves determined by cell counting after infecting with control lentivirus or *PABPC4* knockdown lentivirus. **D.** Representative flow cytometry charts showing early differentiation of early erythroid cells (BFU**-**E) infected with equal amounts of *PABPC4* knockdown or control lentivirus. CD34^-^ CD36^+^ CD71^high^ cells represent the stage of CFU**-**E cells, while CD34^+^ CD36^-^ CD71^low^ cells represent the stage of BFU**-**E cells. CD34^-^ CD36^+^ CD71^high^ and CD34^+^ CD36^-^ CD71^low^ cells were gated from CD45^+^ GPA^-^ CD123^-^ cells. (E) Statistical analysis of the percentage (%) of CD34^-^ CD36^+^ CD71^high^ (CFU**-**E) and CD34^+^ CD36^-^ CD71^low^ (BFU**-**E) cells from three independent experiments is shown. Data shown are mean ± SD (*n* = 3) (*, *P* < 0.05; **, *P* < 0.01; ***, *P* < 0.001).

**Figure S4 Relationship between APA and polyA tail length**

**A.** Density distribution of polyA tail lengths at proximal (turquoise) and distal (coral) polyA sites in control and *PABPC1*_KD, *PABPC1* knockdown samples across three biological replicates. The x-axis represents polyA tail length (nucleotides), and the y-axis shows density. **B.** Comparison of polyA tail length distributions between control group (blue) and *PABPC1*_KD (pink) group at distal (top) and proximal (bottom) PASs. **C.** Gene count analysis showing APA site usage preference (distal *vs*. proximal) associated with changes in polyA tail length. Genes are categorized based on whether they exhibit distal site preference (coral) or proximal site preference (turquoise) across conditions where polyA tails were either lengthened or shortened.

**Figure S5 The verification of APA changes by ONT coverage tracks and qRT-PCR**

ONT coverage tracks of *TSC22D1* (**A**), *PTP4A2* (**B**), *P2RX1* (**C**), and *PARP1*(**D**) comparing control group and *PABPC1*_KD group of erythroid progenitor cells. **E.** The heatmap represents the mean relative expression levels of *TSC22D1* mRNA isoforms utilizing pPASs and dPASs based on qRT-PCR analysis (*PABPC1*_KD group versus control group).

**Figure S6 Western blotting of PABPC1 expression level upon knockdown or overexpression in human induced BFU–E**

**A.** Representative images of western blotting showing PABPC1 expression after *PABPC1* knockdown in human induced BFU**–**E *in vitro*. **B.** Representative images of western blotting showing PABPC1 expression after *PABPC1* overexpression in human induced BFU**–**E *in vitro*.

**Figure S7 *TSC22D1* mRNA expression from GSE61566 and GSE53983**

**A.** The mRNA expression of *TSC22D1* derived from RNA-seq datasets GSE61566 and GSE53983.

**Table S1 APA changes upon *PABPC1* knockdown across different thresholds**

**Table S2 Median polyA tail lengths for genes at pPASs and dPASs across ONT samples**

**Table S3 Comparison of polyA tail lengths between pPASs and dPASs**

**Table S4 Primers used for qRT-PCR**
