# Supplementary material for: Regulation of Alternative Polyadenylation Events by PABPC1 Affects Erythroid Progenitor Cell Expansion
Source: Genomics Proteomics Bioinformatics. 2025 Nov 25;23(6):qzaf116. doi: 10.1093/gpbjnl/qzaf116 (PMC13245397; doi:10.1093/gpbjnl/qzaf116)
Supplement: qzaf116_Supplementary_Data [file qzaf116_supplementary_data.zip › File S1 .docx]

**File S1 The proximal or distal regions of TSC22D1 gene 3' UTR**

**The proximal regions of TSC22D1 gene 3' UTR**

CTGCCTATGCCCCCGCAGAACTGGCTGCTGCGTGTGAACTGAACAGACGGAGAAGATGTGCTAGGGAGAATCTGCCTCCACAGTCACCCATTTCATTGCTCGCTGCGAAAGAGACGTGAGACTGACATATGCCATTATCTCTTTTCCAGTATTAAACACTCATATGCTTATGGCTTGGAGAAATTTCTTAGTTGGGTGAATTAAAGGTTAATCCGAGAATTAGCATGGATATACCGGGACCTCATGCAGCTTGGCAGATATCTGAGAAATGGTTTAATTCATGCTCAGGAGCTGTGTGCCTTTCCATCCCTTCCGGCTCCCTACCCCTCACTTCCAAGGGTTCTCTCTCCTGCTTGCGCTTAGTGTCCTACATGGGGTTGTGAAGCGATGGAGCTCCTCACTGGACTCGCCTCTCTCCTCTCCTCCCCCCAGGAGGAACTTGAAAGGAGGGTAAAAAGACTAAAATGAGGGGGAACAGAGTTCACTGTACAAATTTGACAACTGTCACCAAAATTCATAAAAAACAATAGTACTGTGCCTCTTTCTTCTCAAACAATGGATGACACAAAACTATGAGAGTGACAAAATGGTGACAGGTAGCTGGGACCTAGGCTATCTTACCATGAAGGTTGTTTTGCTTATTGTATATTTGTGTATGTAGTGTAACTATTTTGTACAATAGAGGACTGTAACTACTATTTAGGTTGTACAGATTGAAATTTAGTTGTTTCATTGGCTGTCTGAGGAGGTGTGGACTTTTATATATAGATCTACATAAAAACTGCTACATGACAAAAACCACACCTAAAGAAATTTTAAGAATTTGGCACAGTTACTCACTTTGTGTAATCTGAAATCTAGCTGCTGAATACGCTGAAGTAAATCCTTGTTCACTGAAGTCTTTCAATTGAGCTGGTTGAATACTTTGAAAAATGCTCAGTTCTAACTAATGAAATGGATTTCCCAGTAGGGGTTTCTGCATATCACCTGTATAGTAGTTATATGCATATGTTTCTGTGCATGTTCTCTACACAATTGTAAGGTGTCACTGTATTTAACTGTTGCACTTGTCAACTTTCAATAAAGCATATAAATGTTGATAA**A**

**The distal regions of TSC22D1 gene 3' UTR**

CTGCCTATGCCCCCGCAGAACTGGCTGCTGCGTGTGAACTGAACAGACGGAGAAGATGTGCTAGGGAGAATCTGCCTCCACAGTCACCCATTTCATTGCTCGCTGCGAAAGAGACGTGAGACTGACATATGCCATTATCTCTTTTCCAGTATTAAACACTCATATGCTTATGGCTTGGAGAAATTTCTTAGTTGGGTGAATTAAAGGTTAATCCGAGAATTAGCATGGATATACCGGGACCTCATGCAGCTTGGCAGATATCTGAGAAATGGTTTAATTCATGCTCAGGAGCTGTGTGCCTTTCCATCCCTTCCGGCTCCCTACCCCTCACTTCCAAGGGTTCTCTCTCCTGCTTGCGCTTAGTGTCCTACATGGGGTTGTGAAGCGATGGAGCTCCTCACTGGACTCGCCTCTCTCCTCTCCTCCCCCCAGGAGGAACTTGAAAGGAGGGTAAAAAGACTAAAATGAGGGGGAACAGAGTTCACTGTACAAATTTGACAACTGTCACCAAAATTCATAAAAAACAATAGTACTGTGCCTCTTTCTTCTCAAACAATGGATGACACAAAACTATGAGAGTGACAAAATGGTGACAGGTAGCTGGGACCTAGGCTATCTTACCATGAAGGTTGTTTTGCTTATTGTATATTTGTGTATGTAGTGTAACTATTTTGTACAATAGAGGACTGTAACTACTATTTAGGTTGTACAGATTGAAATTTAGTTGTTTCATTGGCTGTCTGAGGAGGTGTGGACTTTTATATATAGATCTACATAAAAACTGCTACATGACAAAAACCACACCTAAAGAAATTTTAAGAATTTGGCACAGTTACTCACTTTGTGTAATCTGAAATCTAGCTGCTGAATACGCTGAAGTAAATCCTTGTTCACTGAAGTCTTTCAATTGAGCTGGTTGAATACTTTGAAAAATGCTCAGTTCTAACTAATGAAATGGATTTCCCAGTAGGGGTTTCTGCATATCACCTGTATAGTAGTTATATGCATATGTTTCTGTGCATGTTCTCTACACAATTGTAAGGTGTCACTGTATTTAACTGTTGCACTTGTCAACTTTCAATAAAGCATATAAATGTTGATAAACAAGTGTTTTTCATATGACCCTGTTAACATAATGGCAGTCATTTCCACAACTGTTTCCAGGTAAAGTTAACAATTTGACTAGTAAAATCCTGAAGGGCAGTGGAATGATTGGAAAAGTAGGGGCGTGATTTGACTGCATGAAGTAAAAGGAAATTGACACATTGAGTTGTTCAGCAGGTAAGAGAAGAGACAGTGGAGGAGTCCAGCTCAATTCTAACTACTGTGTGACTTGGACAGGTTGCTTCACTCCTGGACGGATGTAGGGGTTTGAAAGAGATGATCTCTTACATTCCCCTTTGGCTTTAAAATTCTAGGAGTCCTTGAAAATCTTAATTTTTTACTGAACTGAGAGGAAGAAGGAGCCTTATTCTGTGTATAATTGGAATAAGCAGATCTAGGATCAAAAGACGGAAGTTGGAGAAGCTGATTTCCACTTTAAGAACCCTGTCACCCTGGGTTGGGATGTTTCTCCTAGTGTAGGATGAGTTTCTAGCTCCTGGGATAATTCGTTTTGTCTAAGCAAAAGATGATTTGAGGTGGGACCAGATGAGCAAGGATGTCCTTGCAGTTGATTCTGCATGACTATAAAACAGGCCAAAATTAAAACCACGAAGGAATCCTGAGGCAGATTGGCCCCTGGCACCTGAGGAGTGAGCCTGAGGGATCCCACTCTGCAGTAGGAGTAACGTGAGCGAGCAAGTGAATGGGGTGGGTTTACCGGCTCTGTAATCTATCAACCTAGGGCCTCAGCAGTGGCCCCCTCCCAGTAGGAATTGGACCAATCCCAAGTTCTAAGGCTTGTCTTAAGTCCTGCAGAACAAGGACAAACTTTCTGAATCATTCATTTCTCTCATCTAGAGCTGGTGGGAGATTGAGAGGTGAATGGGACATCCAAGATCCCTAAAAAGAATTGTTCGATAGCGTGCATGTGTTATAAAGTGGTGACACGGGCATCCTGTTGAAATGATGGATGGCTCACTGCCATAGGCTGATAGCAGTTGTCATAAAGATATTTTGGGGGAATTTGAAAAGGACGTAAAGAAAAATCTTTCATATTGGCTTGTTGGTTATATAACTTCAAATTTAATAAAGGAATACTTACGTAGTAATTACATTTCCTTGAAAAAACTATAGTGAATAGAAATCCCTAGCCATTTCATTTTTTATGTTTTTAATGAAGATCTTTAAAATACCATAGGTGGTAATCGTGAAAATTTGAAAAATCTCATGTCAGTGTATTAAGATGGTGGAGAAGTTTTTTTCTCCATTATTTAATGGAACTTTGGGTCTTTTTATTAAAAATGTGAGACTCATGAA**A**
