# Supplementary material for: Regulation of Alternative Polyadenylation Events by PABPC1 Affects Erythroid Progenitor Cell Expansion
Source: Genomics Proteomics Bioinformatics. 2025 Nov 25;23(6):qzaf116. doi: 10.1093/gpbjnl/qzaf116 (PMC13245397; doi:10.1093/gpbjnl/qzaf116)
Supplement: qzaf116_Supplementary_Data [file qzaf116_supplementary_data.zip › Table S1.docx]

**Table S1 APA changes upon PABPC1 knockdown across different thresholds**

| **Threshold of polyA site usage (PABPC1-KD/control)** | **Distal usage choice** | **Proximal usage choice** |
| --- | --- | --- |
| ± 0 | 3561 | 4049 |
| ± 0.1 | 1761 | 2000 |
| ± 0.15 | 1132 | 1268 |
| ± 0.2 | 766 | 846 |

*Note*: Analysis of APA, alternative polyadenylation changes using ONT-seq, ONT direct RNA sequencing data from erythroid progenitor cells (three control and three PABPC1-KD, PABPC1 knockdown samples). APA analysis was performed using LAPA, a tool specifically designed for third-generation sequencing data.
