# Supplementary material for: Regulation of Alternative Polyadenylation Events by PABPC1 Affects Erythroid Progenitor Cell Expansion
Source: Genomics Proteomics Bioinformatics. 2025 Nov 25;23(6):qzaf116. doi: 10.1093/gpbjnl/qzaf116 (PMC13245397; doi:10.1093/gpbjnl/qzaf116)
Supplement: qzaf116_Supplementary_Data [file qzaf116_supplementary_data.zip › Table S3.docx]

**Table S3 Comparison of polyA tail lengths between proximal and distal polyA sites**

| **Sample ID** | **Proximal site with longer polyA tails** | **Distal site with longer polyA tails** |
| --- | --- | --- |
| Control_1 | 2968 | 3417 |
| Control_2 | 3036 | 3390 |
| Control_3 | 2854 | 3475 |
| PABPC1_KD_1 | 2710 | 3298 |
| PABPC1_KD_2 | 3090 | 3584 |
| PABPC1_KD_3 | 2923 | 3551 |

*Note*: ONT-seq, ONT direct RNA sequencing analysis of the relationship between APA, alternative polyadenylation isoforms and polyA, polyadenylation tail length in erythroid progenitor cells under control and PABPC1_KD, PABPC1 knockdown conditions.
