# Supplementary figures and images for: Regulation of Alternative Polyadenylation Events by PABPC1 Affects Erythroid Progenitor Cell Expansion
Source: Genomics Proteomics Bioinformatics. 2025 Nov 25;23(6):qzaf116. doi: 10.1093/gpbjnl/qzaf116 (PMC13245397; doi:10.1093/gpbjnl/qzaf116)

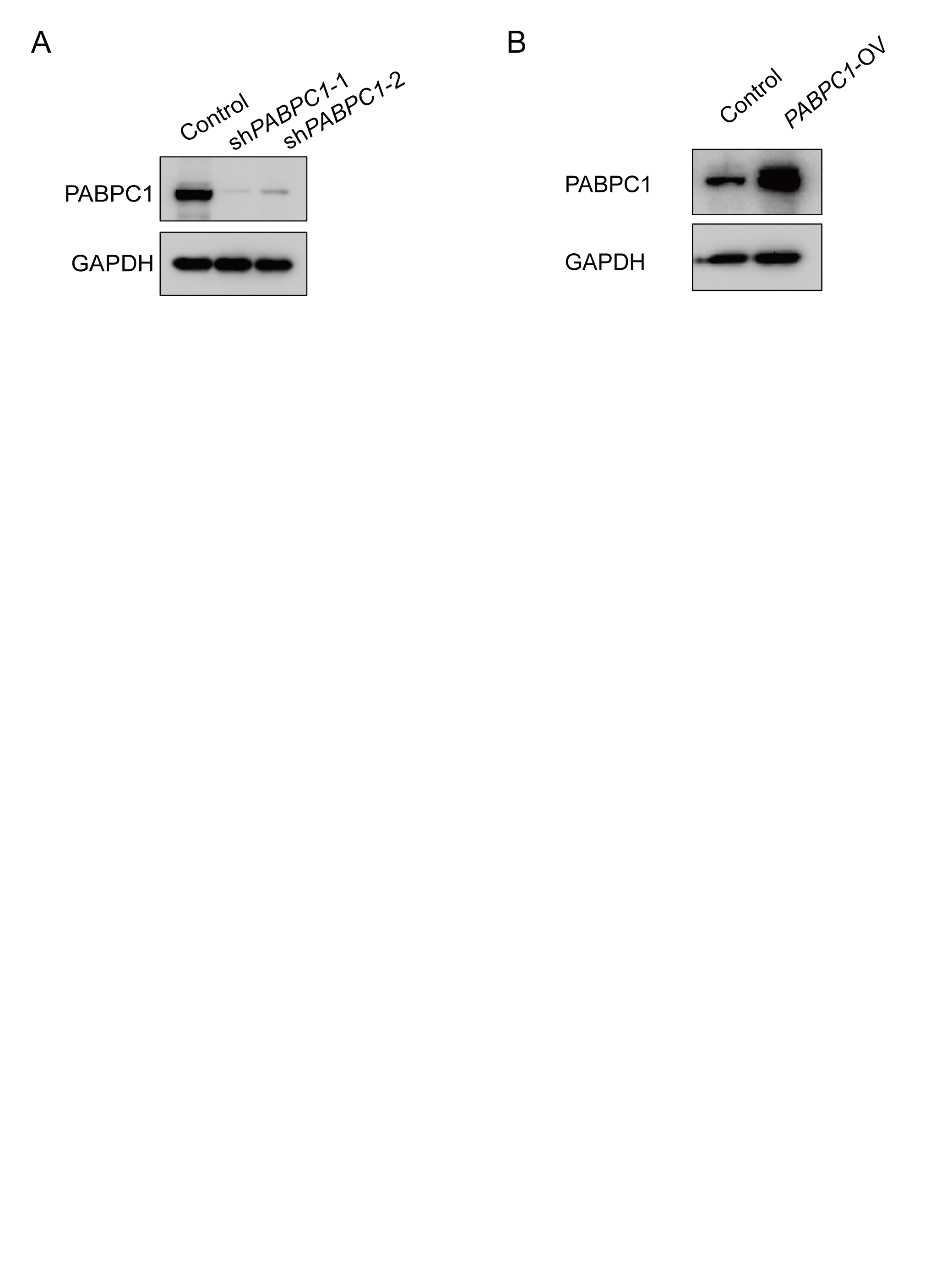

Supplement: qzaf116_Supplementary_Data [file qzaf116_supplementary_data.zip › Figure S6.tif]

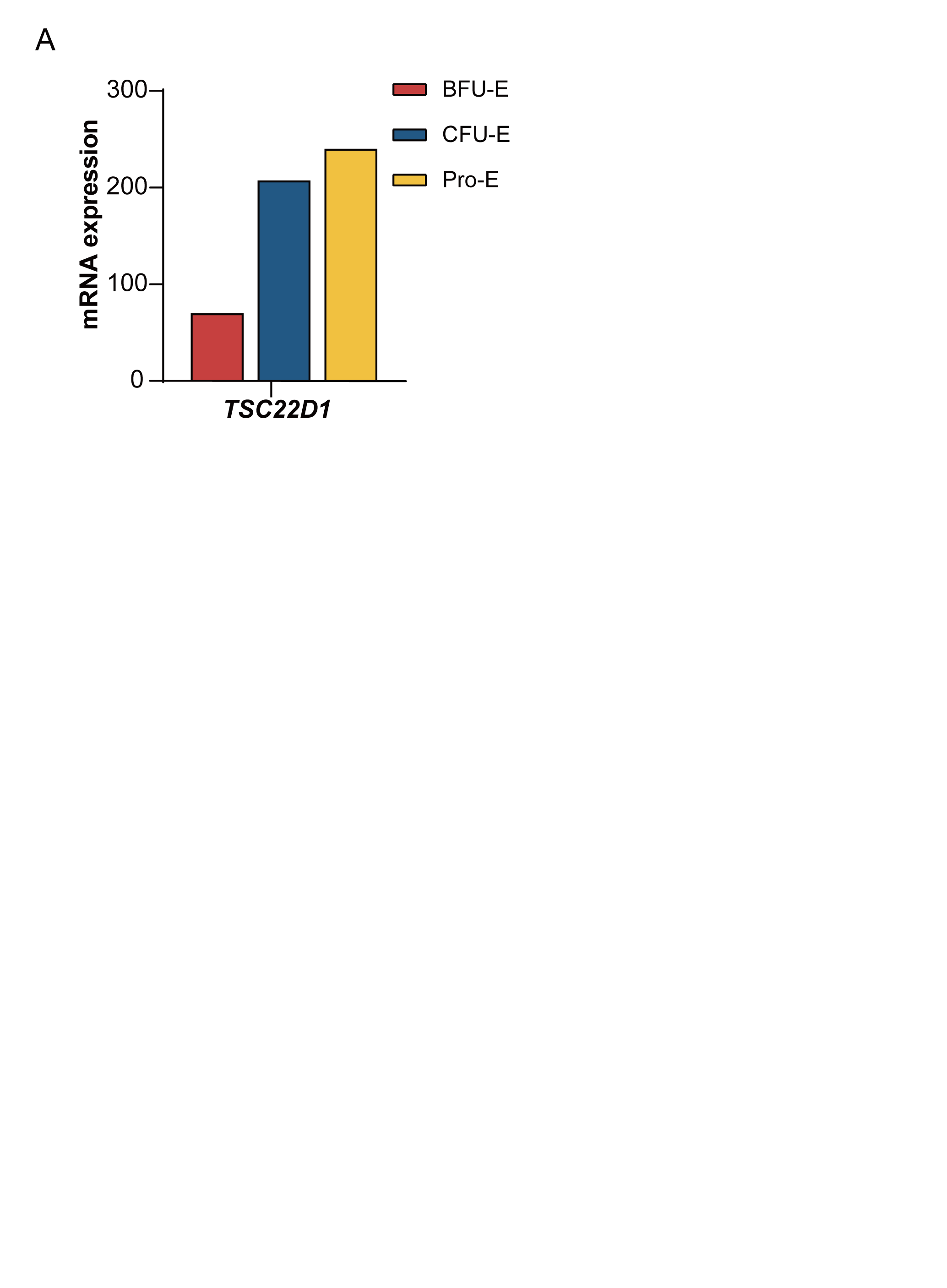

Supplement: qzaf116_Supplementary_Data [file qzaf116_supplementary_data.zip › Figure S7.tif]

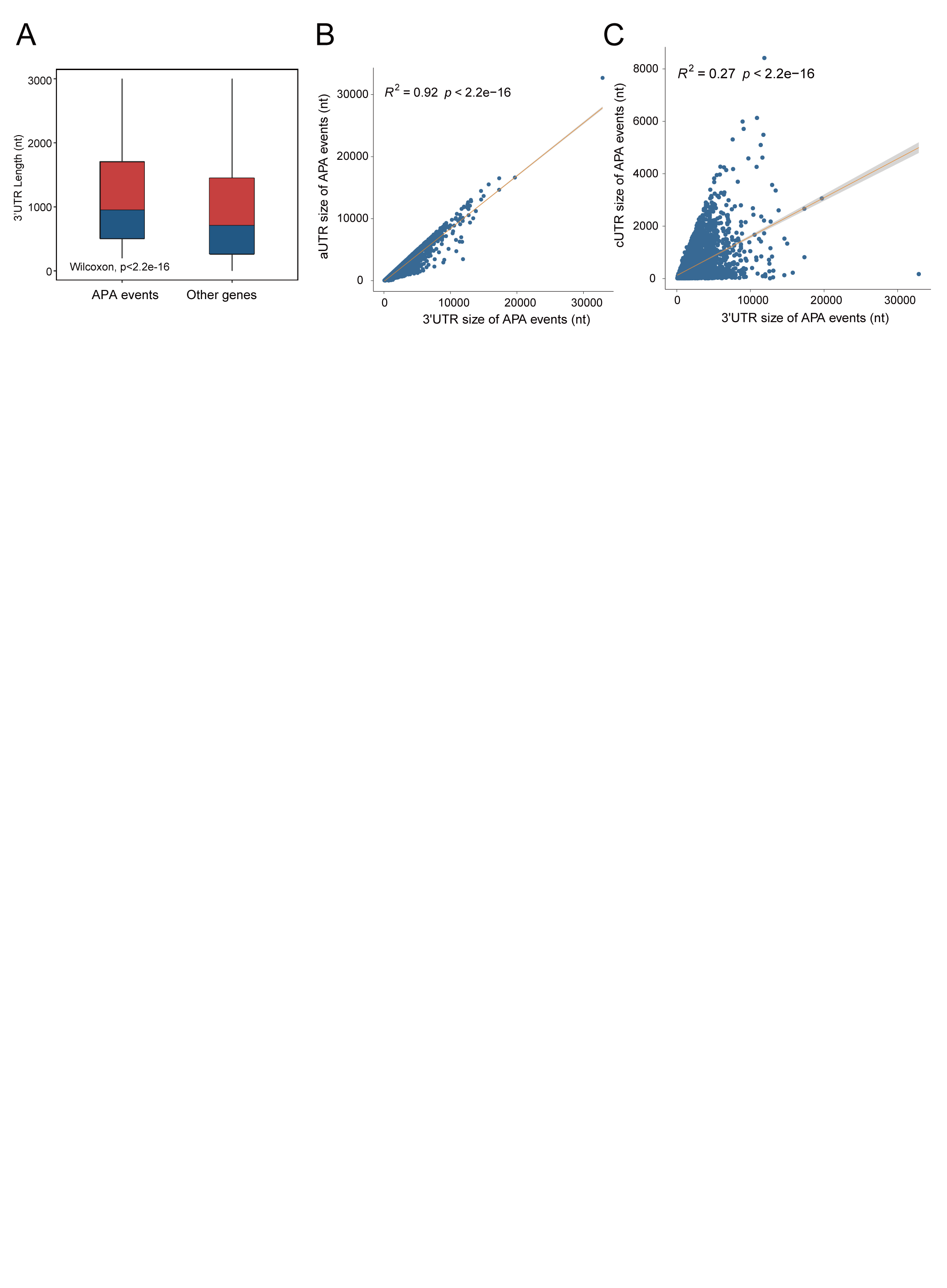

Supplement: qzaf116_Supplementary_Data [file qzaf116_supplementary_data.zip › Figure S1.tif]

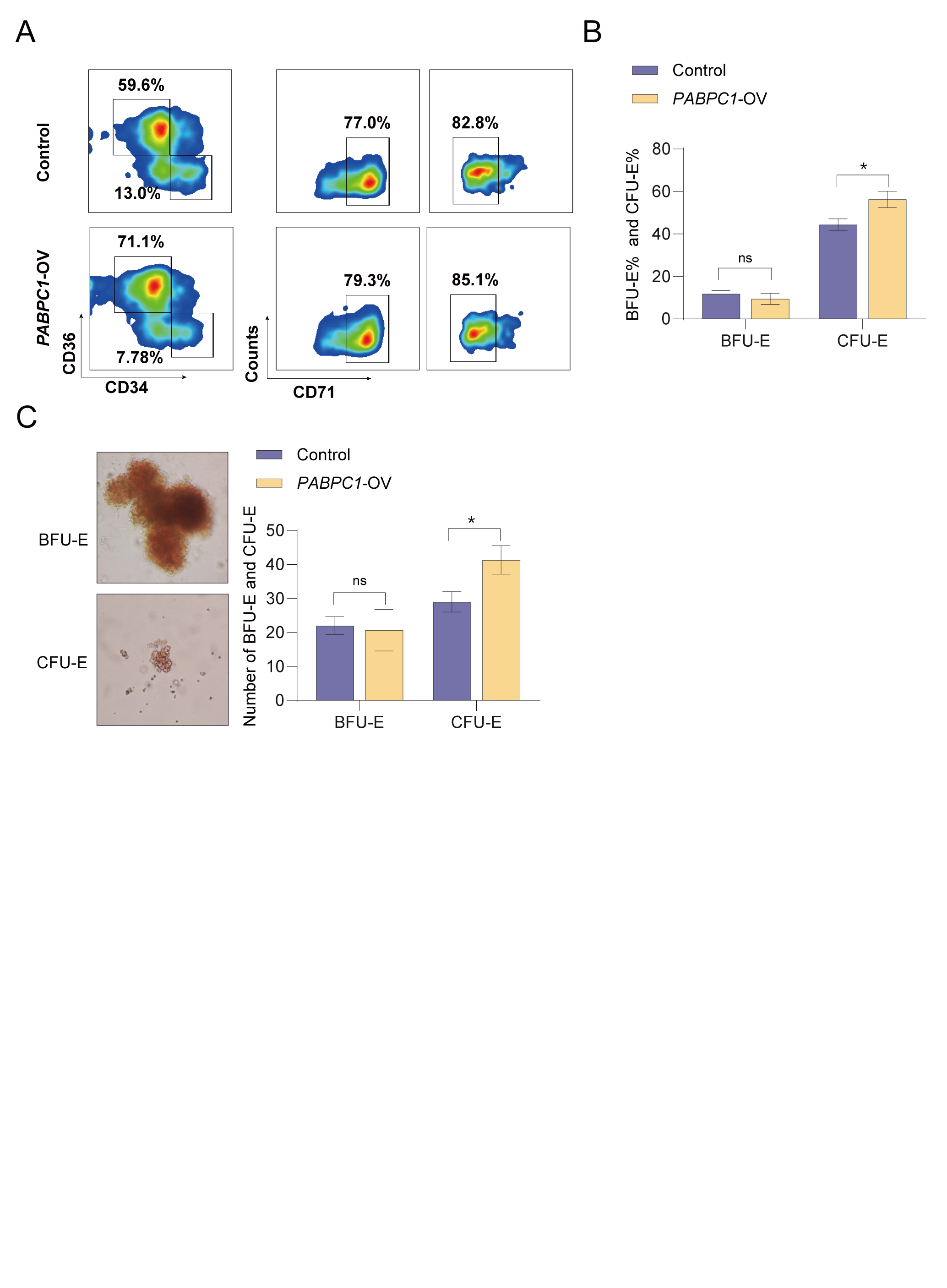

Supplement: qzaf116_Supplementary_Data [file qzaf116_supplementary_data.zip › Figure S2.tif]

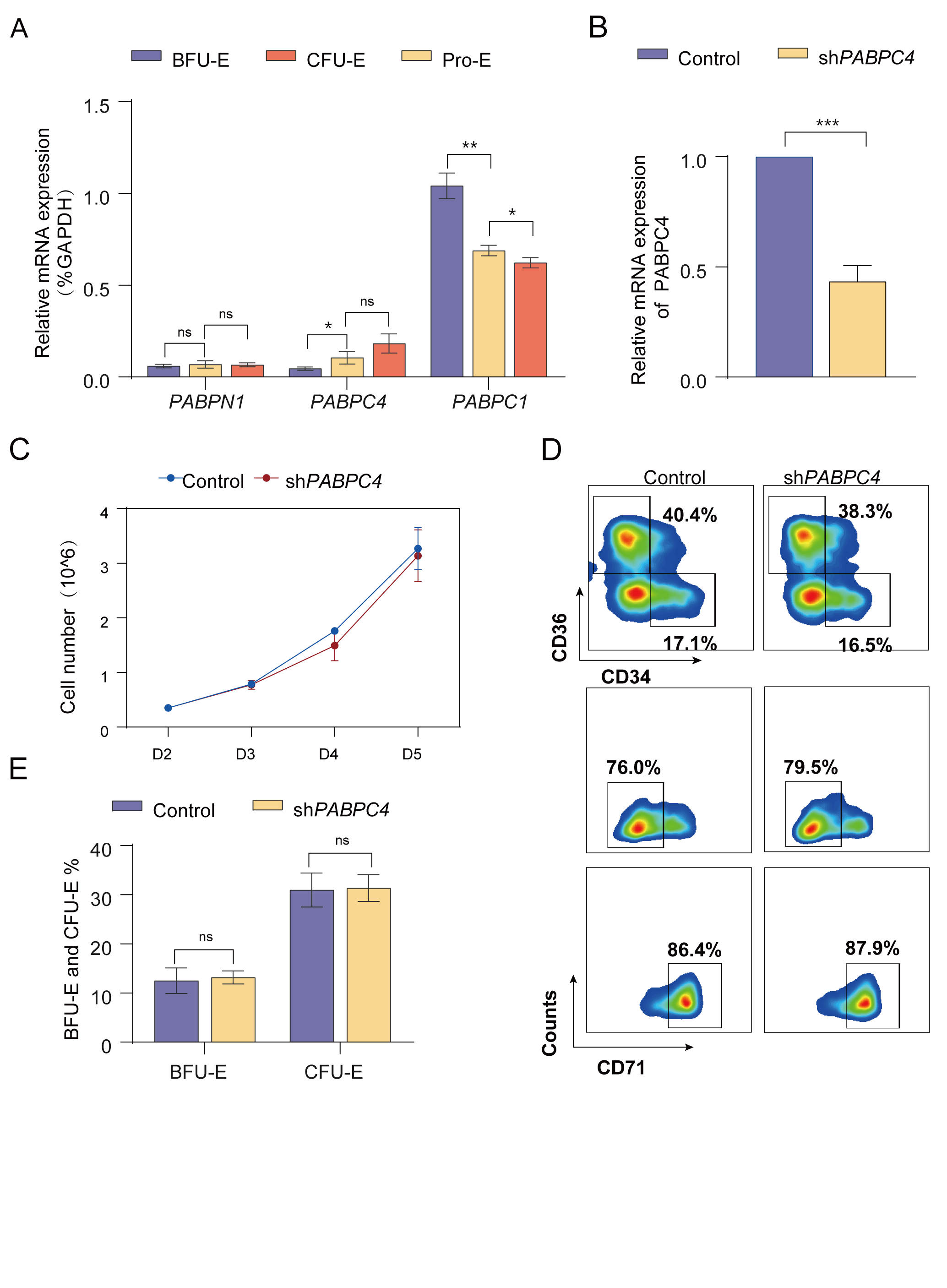

Supplement: qzaf116_Supplementary_Data [file qzaf116_supplementary_data.zip › Figure S3.tif]

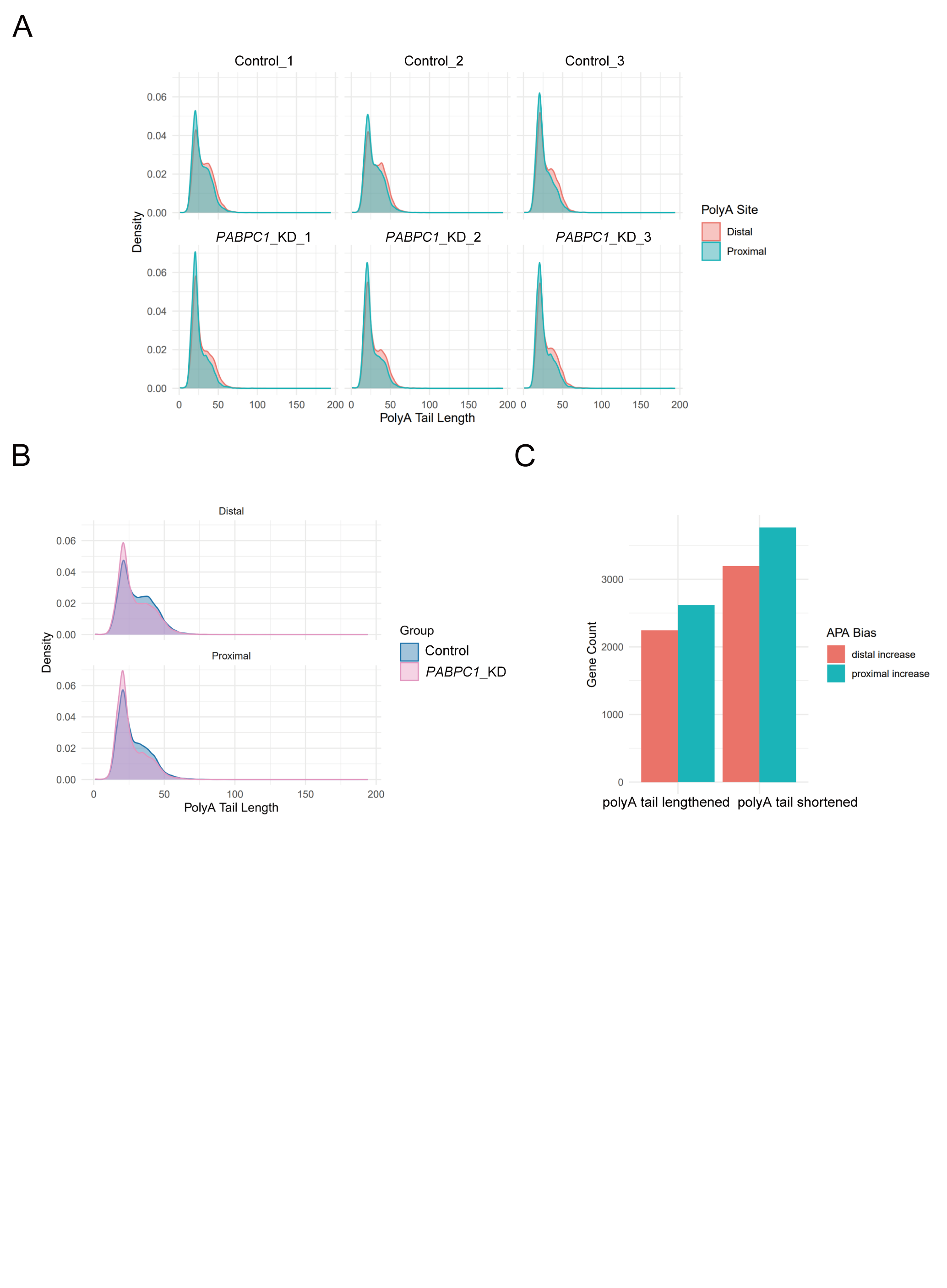

Supplement: qzaf116_Supplementary_Data [file qzaf116_supplementary_data.zip › Figure S4.tif]

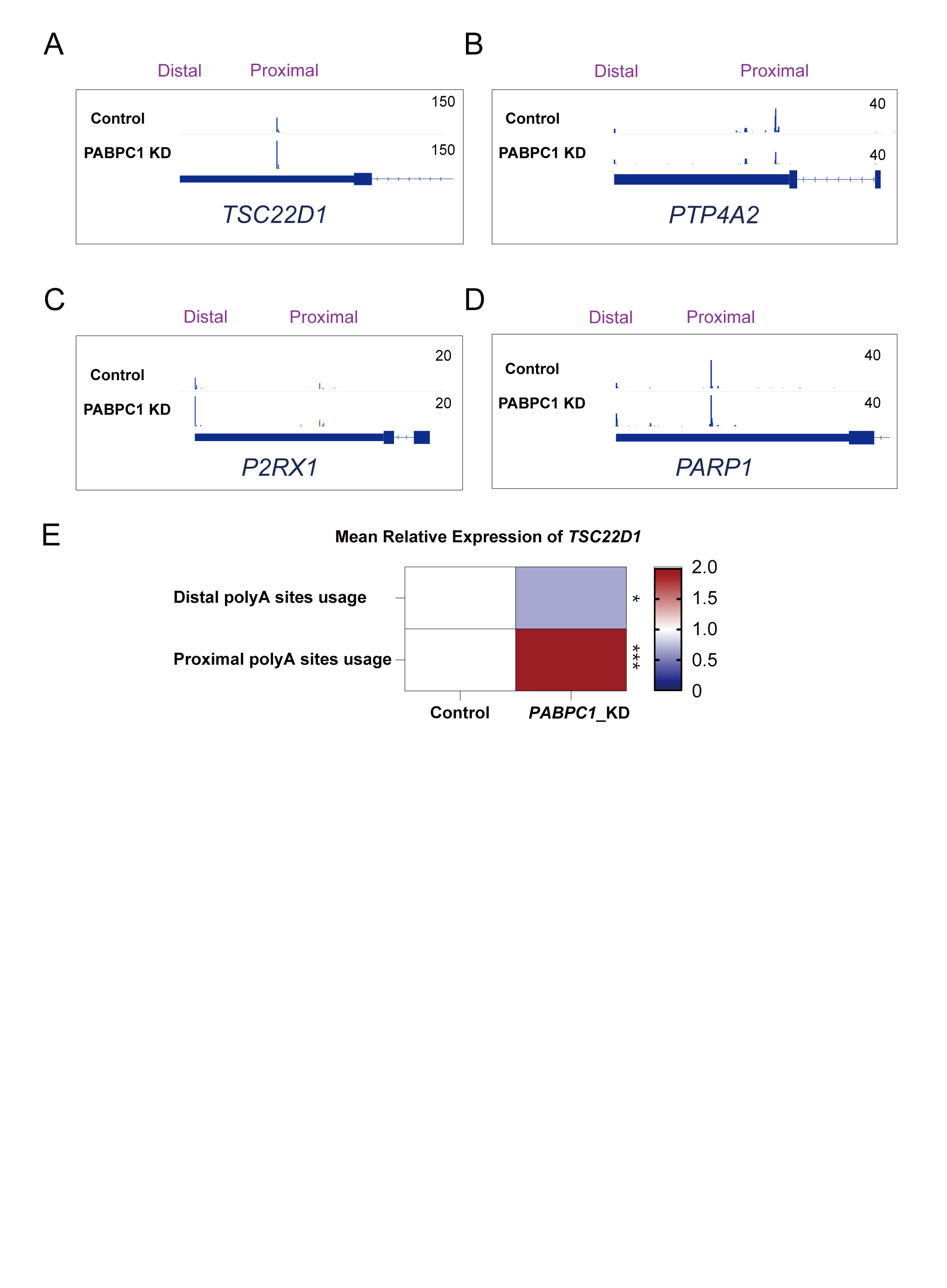

Supplement: qzaf116_Supplementary_Data [file qzaf116_supplementary_data.zip › Figure S5.tif]
